# Poly(A) RNA sequencing reveals age-related differences in the prefrontal cortex of dogs

**DOI:** 10.1007/s11357-022-00533-3

**Published:** 2022-03-14

**Authors:** Sára Sándor, Dávid Jónás, Kitti Tátrai, Kálmán Czeibert, Eniko Kubinyi

**Affiliations:** 1 Department of Ethology, ELTE Eötvös Loránd University, 1/c Pázmány Péter sétány, Budapest, 1117 Hungary; 2 Department of Genetics, ELTE Eötvös Loránd University, 1/c Pázmány Péter sétány, Budapest, 1117 Hungary

**Keywords:** RNA sequencing, Dog, Aging, Prefrontal cortex

## Abstract

Dogs may possess a unique translational potential to investigate neural aging and dementia because they are prone to age-related cognitive decline, including an Alzheimer’s disease–like pathological condition. Yet very little is known about the molecular mechanisms underlying canine cognitive decline. The goal of the current study was to explore the transcriptomic differences between young and old dogs’ frontal cortex, which is a brain region often affected by various forms of age-related dementia in humans. RNA isolates from the frontal cortical brain area of 13 pet dogs, which represented 7 different breeds and crossbreds, were analyzed. The dogs were euthanized for medical reasons, and their bodies had been donated by their owners for scientific purposes. The poly(A) tail RNA subfraction of the total transcriptome was targeted in the sequencing analysis. Cluster analyses, differential gene expression analyses, and gene ontology analyses were carried out to assess which genes and genetic regulatory mechanisms were mostly affected by aging. Age was the most prominent factor in the clustering of the animals, indicating the presence of distinct gene expression patterns related to aging in a genetically variable population. A total of 3436 genes were found to be differentially expressed between the age groups, many of which were linked to neural function, immune system, and protein synthesis. These findings are in accordance with previous human brain aging RNA sequencing studies. Some genes were found to behave more similarly to humans than to rodents, further supporting the applicability of dogs in translational aging research.

**Supplementary Information:**

The online version contains supplementary material available at 10.1007/s11357-022-00533-3.

## Introduction

Several studies have suggested dogs as promising models for aging [1–4] because dogs have a much shorter lifespan than humans, yet undergo a highly similar aging course and are prone to develop analogous age-related pathologies. Importantly, a proportion of old dogs develop canine cognitive dysfunction (CCD), which shares many similarities with human Alzheimer’s disease (AD), in both symptoms and brain pathology [5–7]. Dogs, therefore, may gain a key role in dementia research, as this form of age-related neurodegeneration does not naturally occur in most laboratory model organisms, including rodents. This is a highly relevant area of research, as most drugs developed to treat Alzheimer’s disease or other forms of dementia have failed human clinical trials even if they seemed efficient in mice suffering from artificially induced dementia [8]. Finding an appropriate animal model with a higher predictive capacity would greatly augment the development of drugs and other forms of interventions.

Laboratory dogs have already been traditionally used as preclinical models to test drugs, and many studies have investigated aspects of brain aging in laboratory dog populations [9, 10]. However, keeping laboratory dogs for aging and dementia research would not necessarily be a cost-effective solution as the spatial, time, and financial requirements greatly exceed the needs of smaller laboratory animals. In addition, if the goal is to observe naturally occurring dementia, the need for time and the number of involved animals would increase dramatically, as CCD symptoms occur in 14.4–18% of dogs above the age of 14 years [11, 12]. In contrast, pet dogs are numerous and can also have additional benefits compared to laboratory dogs.

Most pet dogs live as companions of their owners and therefore are exposed to similar environmental stressors, which could strongly affect aging and age-related diseases. Variances in the diet and lifestyle of pet dogs could also be analogous to the variances found among their owners [13–16]. Pet dogs come from a large variety of breeds and therefore show a huge natural genotypic and phenotypic variability, including a wide range of expected lifespan. The life expectancy of dogs, in general, is associated with their average body weight, as smaller dogs tend to live longer [17–20].

One factor suspected to be responsible for this difference is the insulin-like growth factor 1 (IGF1) signaling pathway, which is known to regulate both body size and lifespan across animals [21–24]. Size-determining polymorphisms in IGF1 signaling–related genes have already been described in dogs [25, 26]. However, it is highly plausible that a range of other mechanisms also contributes to the observed correlations between body size, breed, and expected lifespan and these are still unexplored in dogs [27]. These factors can be unraveled by next-generation high-throughput genomic approaches.

The high genetic variability and unique population structure within and between dog breeds have already prompted the species to become a good target for genetic investigations [28–30]. So far, next-generation sequencing and microarray technology have been successfully applied to detect genomic variants responsible for qualitative phenotypic variation among breeds and quantitative trait loci have also been successfully mapped [31–34]. To date, the intricate details of the genetic regulatory mechanisms affecting complex phenotypes and disease pathology are less explored in dogs, partly because these investigations must rely on cellular RNA and protein content from different tissues. Tissue samples derived from laboratory dogs do not suffice for this goal as these dogs do not represent the natural phenotypic variation of the species. On the other hand, it is challenging to collect tissue samples from companion dogs as this would usually require invasive approaches. Some recent initiatives of dog transcriptomic investigations have relied on canine biobanks to collect tissues from companion dogs postmortem [35–38].

In humans, RNA sequencing has greatly contributed to the understanding of various complex conditions and diseases, including aging [39–42] and age-related diseases, such as different forms of cancer [43, 44] and neurodegeneration [45–48]. Importantly, RNA sequencing technology has allowed researchers to explore several aspects of the transcriptomic landscape, which would remain hidden using microarray technology [49]. For example, alternative splicing events could only be detected by microarray if the mapping points are in different exons of a messenger RNA (mRNA) and the changes in the ratio of the exons are large enough to be detectable. RNA sequencing offers a direct approach to investigate alternative splicing. An increasing body of evidence suggests that aging is generally associated with changes in splicing activity in both humans and model organisms [50, 51]. Different splice variants of a gene may play varying roles in divergent regulatory pathways. Changes in their ratio could affect fundamental cellular processes, such as sugar-sensing pathways and regulation of gene expression. Additionally, more and more findings suggest that aberrant alterations in the splicing of several genes are strongly associated with Alzheimer’s disease [52, 53]. Altogether, the in-depth analysis of transcriptomic changes allowed a more detailed insight into the intermittent mechanisms underlying aging and neurodegeneration in humans. Both shared and distinct patterns were identified between healthy aging and dementia [52, 54], which is a major step towards developing predictive methodologies and interventions. As the primate brain and especially the human brain have several unique developmental attributes regulated by lineage-specific genetic factors [55–57], it is not self-evident that dogs’ dementia is entirely analogous to its human counterpart at the level of genetic regulatory networks. For exploring the evolutionary conservation of the regulatory network alterations responsible for neural aging, we need highly detailed transcriptomic data from the affected canine brain regions.

The frontal cortex is a region that shows well-characterized and distinct cellular [58, 59], ultrastructural, and molecular changes with aging in humans [60, 61] and is also primarily affected in various forms of dementia [62]. Distinct cellular and ultrastructural changes in the frontal cortex were also reported in dogs in relation to aging and CCD [63, 64]. However, only a few studies investigated the associated molecular changes, e.g., the expression levels of genes linked to oxidative stress [65]. So far, the most detailed gene expression analysis between young and old dogs’ cerebral cortices was done using Affymetrix GeneChip® Canine Genome Arrays [66]. Although this study represented an important step in exploring age-related changes in the canine brain, the applied technology could not cover all the relevant aspects of the transcriptomic changes. Furthermore, the exclusive inclusion of beagle dogs in the cited study may reduce the generalizability of the results. To the best of our knowledge, the age-related differences in the transcriptomic profile in the frontal cortex of companion dogs, representing the natural genetic variability of the species, have not been explored yet.

With this in mind, we investigated the complete mRNA set in the frontal cortices of six young and seven old dogs, which represented various breeds with medium to large body sizes. We applied poly(A) capture RNA sequencing to gain insight into the age-related changes in the expression of protein-coding genes in the dog brain. We then compared our list of differentially expressed genes with previous human and mouse studies, which also investigated the frontal cortical area of the brain, to determine the ratio of shared genes. Such comparisons could allow a better view of the translatability between the three species and can clarify whether dogs play a gap-filling role in aging and dementia research between mice and humans. In addition, based on previous human studies, we investigated the age-related alterations in splicing patterns, as alternative splicing also represents a major genetic regulatory mechanism.

## Methods

### Subjects

This study included 6 young dogs (aged 1–4 years; average body weight 13.3 kg ± 5.4 kg; breeds: 3 beagles, 1 boxer, 1 border collie, 1 German shepherd dog; sex: all females) and 7 old dogs (aged 14–17 years; average body weight 21.4 kg ± 6.3 kg; 3 mixed-breeds, 1 border collie, 1 golden retriever, 1 small Münsterländer, 1 Hungarian Vizsla; sex: 5 females, 2 males) (Table 1). All dogs had been euthanized for medical reasons. The cadavers of the animals were donated by their owners to the *Canine Brain and Tissue Bank* [38]. This biobank contains the brains and other tissue samples (e.g., fur, muscle) together with medical reports and behavioral test results (if available) of the dogs, which had been offered by their owners for scientific purposes. Since the biobank relies on euthanized companion dogs, samples could not be systematically collected to represent the same breeds in both the young and old cohorts. To reduce heterogeneity, dogs from the middle-large body size category were selected for sequencing, excluding giant and small breeds from the current analysis. Based on previous studies [67, 68], the sample size used is appropriate for differential gene expression analysis. To assess the within-breed variance of gene expression as a baseline for the expected inter-individual variance, three beagles were included in the young group.

**Table 1:** Subjects included in the study

Age group	Animal ID	Abbreviation in MDS figures	Breed	Age (years)	Sex	Body mass (kg)
**Young**	CL_eto1	GSD	German shepherd dog	4	Female	20.2
	CL_eto2	Be1	Beagle	3	Female	10.3
	CL_eto3	Be2	Beagle	3	Female	11.2
	CL_eto4	BC1	Border collie	1	Female	10.7
	CL_eto5	Bo	Boxer	1	Female	20.1
	CL_eto6	Be3	Beagle	3	Female	7.5
**Old**	CL_eto7	MB1	Mixed-breed (German shepherd dog)	14	Female	29.7
	CL_eto8	BC2	Border collie	14	Female	13.2
	CL_eto9	GR	Golden retriever	17	Female	22.0
	CL_eto10	SM	Small Münsterländer	17	Female	12.7
	CL_eto11	MB2	Mixed breed	14	Female	25.4
	CL_eto12	Vi	Vizsla	15	Male	25.0
	CL_eto13	MB3	Mixed-breed (border collie-mudi)	14	Male	21.5

The 2 age groups were very distinct, with dogs aged 1–4 years in the young group and dogs aged 14–17 years in the old group (Table 1). The wide age gap of 10 years between the age groups is approximately 73% of the overall average dog lifespan [19].

### Frontal cortex sample collection

Brain tissue collection took place within 4 h postmortem. The brains were removed from the skull intact, rinsed in room-temperature phosphate-buffered saline, and immediately processed for sample collection and storage. Small pieces (~ 100 mg) of the cortical area were cut from the frontal lobe and immersed in RNAlater (Thermo Fisher Scientific). Samples were transferred to a − 80 °C ULT freezer after being kept at 4 °C to ensure complete penetration of the stabilizing agent, which was then removed as supernatant, leaving only the tissue pieces in the tubes.

### RNA extraction

Total RNA content was extracted from RNAlater-stabilized brain tissue samples using the RNeasy Mini Kit (Qiagen), following the manufacturer’s protocol. RNA samples were dissolved in RNase-Free Water (AccuGENE™ Molecular Biology Water (Lonza)) and were stored at − 80 °C until further processing.

### Library preparation and sequencing

All further steps from RNA isolation to sequencing were carried out by Omega Bioservices (Norcross, Georgia, USA). RNA integrity number (RIN) numbers were determined by an Agilent 2100 Bioanalyzer, and all samples met the required quality criteria (RIN > 7.9). RNAs with poly(A) tail were selected prior to library preparation using the poly(A) capture method. Libraries were then prepared following the Illumina’s TruSeq Stranded mRNA library preparation protocol (Illumina). Finally, the libraries were sequenced at Omega Bioservices (Norcross, Georgia, USA).

### Sequence data analysis

Illumina TruSeq adapters were trimmed with the *Cutadapt* script [69], using the following options: the -a and -A options to provide the adapter sequences for the forward and reverse reads, the -m option to set the minimum length of retained reads to 50 bp, the -j option to run the adapter trimming on five processors in parallel, and the -o and -p options to set the output files for the forward and reverse reads, respectively. Following adapter trimming, the raw sequence data quality was assessed with FastQC software [70], using the default parameters.

The HISAT2 v2.0.0 [71] aligner was used in paired-end mode to align the raw reads to the reference genome (reference genome version: CanFam v3.1). In addition to the obligatory parameters (-x; -1; -2; -S), the following options were used during the alignment: the -p option to set the number of processors used for alignment to six instead of the default one and the --dta option so the output can be used by transcript assemblers. Following sequence alignment, some standard post-alignment steps were implemented on the obtained SAM files, including compression to the BAM file format, sorting, indexing (all done with the Samtools software suite [72]), and the creation of tdf files for Integrative Genomics Viewer (IGV) visualization (IGVTools [73]). Alignment quality was also assessed by calculating some alignment statistics (BAMTools [74]).

Next, the RNA-Seq analysis pipeline described in Pertea et al. [76] was implemented, except for the final step. This pipeline included a per-sample transcript assembly step using the StringTie v2.1.2 software [77] (options used were as follows: -G with the genome annotation file (gtf format) downloaded from Ensembl release 98 and -o to provide an output file), a merger of the per-sample data files into a single file that included all transcript abundance information from all 13 individuals (StringTie software with the --merge option; otherwise, the same parameters were used as previously with StringTie). In a third StringTie run, the -B and -e options were used as well to create the proper input files for the *Ballgown* R package. In the final step of this pipeline, the analysis with the Ballgown R package was replaced with an analysis using the *edgeR* package (v3.24.3; similar to Megquier et al. [36]). The Ballgown input file format was transformed to the file format required by edgeR using the prepDE.py script provided with the StringTie software. edgeR was used to run the differential gene expression analysis on our samples. Finally, data was interpreted, and tables and figures were created mainly in R and Microsoft Office (Excel). Gene ontology analysis was performed using the PantherDB (v16.0) online tool available at the http://www.pantherdb.org website and which is part of the *Gene Ontology Phylogenetic Annotation Project* [78, 79].

**Fig. 1:**
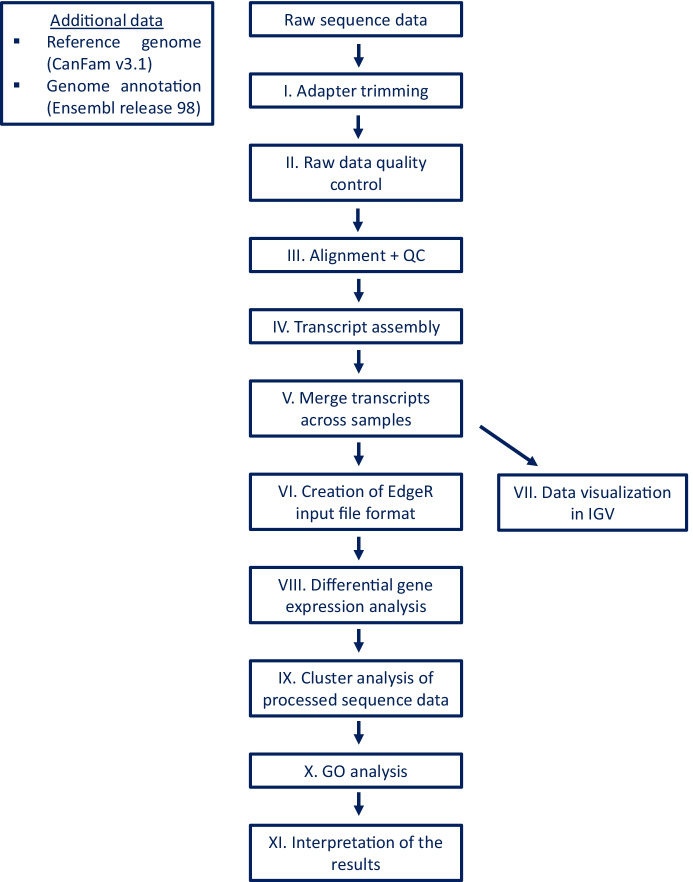
The simplified analysis pipeline used for data analysis

A summary of the applied analysis pipeline is shown in Fig. 1.

### Statistical methods

#### Multidimensional scaling analysis

Multidimensional scaling was performed using both pair-wise differences in log-transformed fold changes of all expressed genes and the biological coefficient of variation to estimate sample distances. Once the sample distances were estimated, figures were created using the ggplot2 R package [80].

#### Gene ontology analysis

In the gene ontology analysis, a regular statistical overrepresentation test was implemented using the Panther classification system’s online tool. The set of all differentially expressed genes was used as the target list, and the collection of all genes expressed in our samples was used as the reference list for this analysis. Primarily, the PantherDB’s slim gene ontology (GO) annotation set was used for the analysis. However, to allow a more detailed discussion, the complete gene ontology database was applied as well.

#### Splicing analysis

Alternative splicing events were examined as described in Schafer et al. [81]. In short, the frequency of the splicing events at any particular splice site was estimated using the number of split reads overlapping with the splice site but excluding one of the exons (exclusion rate (ER)) and those that include the same exon (inclusion rate (IR)). Following some standard normalizations (e.g., for the read length and the exon length), the percent spliced in index (PSI) can be calculated as ${\mathrm{PSI}}_{i}=\frac{{\mathrm{IR}}_{i,\mathrm{n}}}{{\mathrm{IR}}_{i,\mathrm{n}}+{\mathrm{ER}}_{i,\mathrm{n}}}$, where PSI_*i*_ is the estimated PSI of exon *i*, and IR_*i*,n_ and ER_*i*,n_ are the normalized inclusion and exclusion rates for exon *i*, respectively. A PSI value of 1 indicates an exon that was included in all sequenced transcripts, while a PSI lower than 1 indicates that the exon was included only in a fraction of the transcripts derived from that particular gene (e.g., 0.1 indicates that the exon was included in 10% of the transcripts). For further details on the method, see Schafer et al. [81]. The analysis was performed using primarily in-house scripts.

### Investigating the effect of possible blood content

To investigate the extent of possible “contamination” by residual blood in the brain tissue, the expression levels of four transcripts, the human homologs of which have been reported to be highly expressed in red blood cells [82], were checked. These transcripts were the following: ENSCAFG00000032615, ENSCAFG00000029224, ENSCAFG00000028569, and ENSCAFG00000030286.

### Real-time qPCR

One microgram of each total RNA isolate was reverse-transcribed into complementary DNA (cDNA) using the RevertAid First Strand cDNA Synthesis Kit (Thermo Fisher Scientific) following the manufacturer’s protocol. cDNA samples were diluted 10 × and were stored at − 20 °C before subsequent measurements. RT-qPCR was performed on a Roche LightCycler 96 instrument. Each reaction was run in duplicate. Detection of amplification was done using the PowerUp SYBR Green Master Mix (Thermo Fisher Scientific) reagent with primer pairs specifically designed for the chosen target genes. GAPDH was used as house-keeping gene, and the ΔΔCt method was applied to normalize and compare the expression levels of target genes. Gene list and applied primer sequences can be found in Table S1.

## Results

On average, 74 million reads were sequenced per sample (ranging from 56.2 million to 103.3 million), an average of 97% of which could be aligned to the reference genome. The quality of the raw (i.e., unaligned sequence) data was investigated with the FastQC software, and no problematic issues were encountered other than some adapter sequence contamination in the sequences, which were trimmed. This resulted in a reduced read length when compared to the original sequencing length (150 bp), although the shortened reads were still 124 bp long on average (Table 2). The 97% alignment rate is exceptionally high; as a comparison, Yang et al. [83] observed an 88% alignment rate, when they aligned short reads to the same reference genome. The large sequencing depth with the observed high alignment rate ensured that even rare transcripts could be detected in our dataset. Altogether, we found 16,071 genes with ~ 38,000 transcripts to be expressed in at least one sample in our dataset.

**Table 2:** Per-sample sequence information. The “Number of sequenced reads” and the “Number of aligned reads” are rounded to the nearest integer

Animal ID	Number of sequenced reads	Sequenced bases (GB)	Average read length (bp)	Aligned reads	
				***N***	**%**
**CL_eto1**	78,444,190	9.82	125.25	76,327,626	97.30
**CL_eto2**	65,698,972	8.16	124.23	63,724,440	96.99
**CL_eto3**	60,778,246	7.65	125.95	58,800,613	96.75
**CL_eto4**	75,120,464	9.29	123.73	73,040,932	97.23
**CL_eto5**	56,204,562	6.96	123.83	54,602,324	97.15
**CL_eto6**	71,834,902	8.97	124.81	69,512,254	96.77
**CL_eto7**	70,806,132	8.68	122.64	68,866,039	97.26
**CL_eto8**	73,649,554	9.09	123.48	71,708,819	97.36
**CL_eto9**	103,255,050	12.60	122.01	100,561,881	97.39
**CL_eto10**	73,830,824	9.05	122.59	71,354,911	96.65
**CL_eto11**	91,376,292	11.19	122.46	88,906,853	97.30
**CL_eto12**	78,653,918	9.62	122.25	76,869,045	97.73
**CL_eto13**	62,258,664	7.80	125.31	60,557,001	97.27
**Average**	73,993,213	9.15	123.73	71,910,211	97.17

### Age groups were separated based on gene expression

**Fig. 2:**
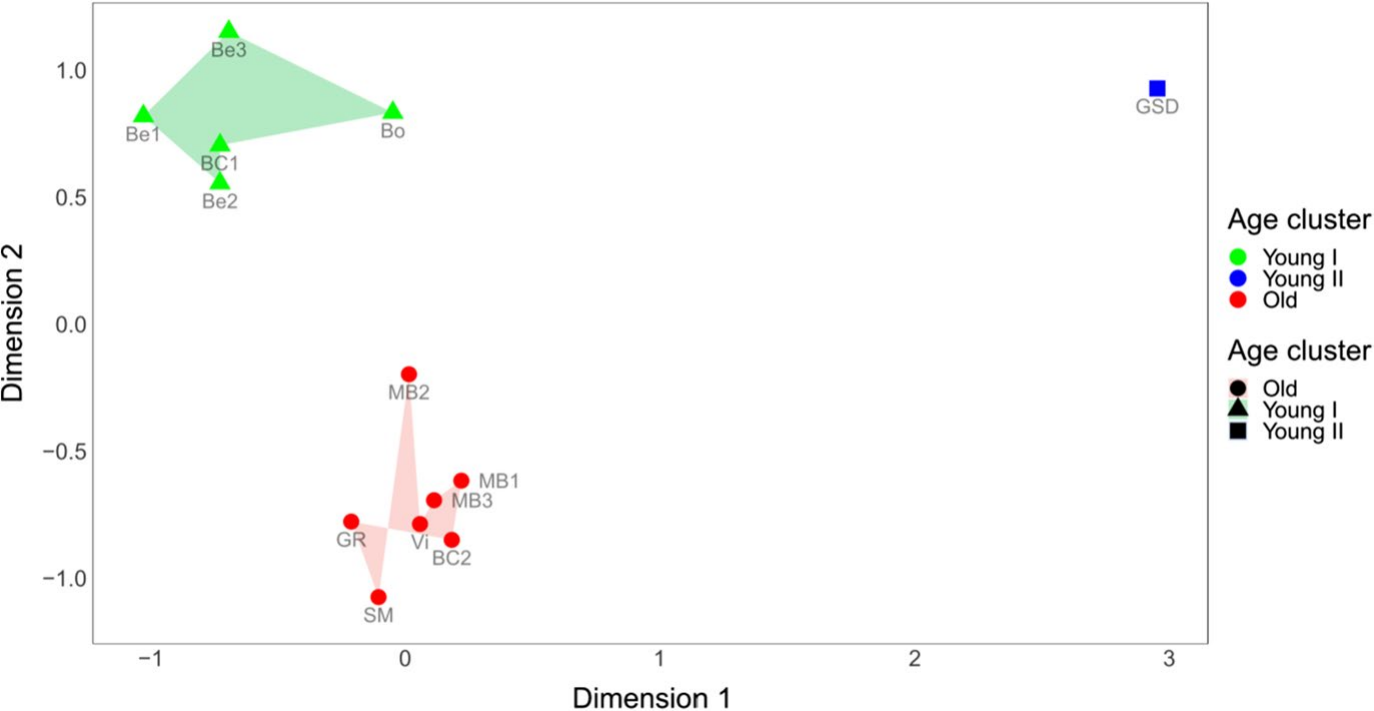
Multidimensional scaling of the CPM (counts per million reads) values for each gene expressed in the samples. Distances between the individuals represent the pairwise differences in log-transformed fold changes. Coloring of the groups is according to the visibly distinguishable clusters. Abbreviations belonging to each animal have been included in Table 1

Prior to the differential gene expression analysis, a multidimensional scaling (MDS) of the counts per million (CPM) sequenced reads was implemented (Fig. 2). Three clusters could be differentiated on this plot: the old and young individuals were separated explicitly along the dimensions, while one animal showed a position detached from both groups. Importantly, the three beagle dogs included in the young cohort did not show any substantial clustering with each other within the young group, especially in relation to CL_eto4, which was a border collie. This was further supported by the differential gene expression analysis (see below).

The outlier animal, Cl_eto1, which did not fall within either the young or old cluster, was a 4-year-old German shepherd dog. In the MDS analysis, this individual was very similar to the other young animals along with dimension #2, while it was clearly separated from both cohorts along with dimension #1, with a somewhat smaller distance from the old cohort.

A second multidimensional scaling analysis, where the biological coefficient of variation of the top genes (*n* = 500) was used instead of the *log*_*2*_* fold change* distances, gave very similar results (see Fig. S1), including the separation of CL_eto1 from both cohorts. However, in this case, a greater distance was observed from the young cohort along dimension #2. Altogether, this individual seemed to show a somewhat intermediate gene expression level between the young and old cohorts. This may be consistent with the notion that this individual was the oldest among the young animals (at 4 years old) and belonged to a larger breed, i.e., to a breed with a shorter average lifespan. Other than this German shepherd dog, the dogs were clustered according to their age cohort and irrespective of their body size, sex, or other parameters.

Based on these results, we decided to exclude CL_eto1 from the differential gene expression analysis presented below. However, the results of the differential gene expression analysis including all animals are shown in the supplementary materials of the article. Further supporting evidence for the exclusion of CL_eto1 is also presented in the “Exclusion of the extreme outlier (CL_eto1)” section of the “Supplementary document” and in Table S2/Fig. S2 of the supplementary materials.

As only two male dogs were included in the study and both were in the old age group, additional differential gene expression analyses were performed with either excluding these two animals or excluding two other, randomly chosen old animals (in settings where CL_eto1 was already excluded). This way, the extent of the possible bias caused by two males included only in the old cohort could be estimated. The number of altered differentially expressed genes (DEGs) were comparable between the “two-males” and “two-random animals” settings (Table S3), and the list of genes, which uniquely changed in the two-male setting, did not show any GO enrichment (e.g., related to sex). Also, the positioning of these two animals in the MDS plots (Fig. 2, Fig. S1) suggested that sex difference had no major effect on gene expression patterns in this investigation.

### DEGs in young and old dogs

**Fig. 3:**
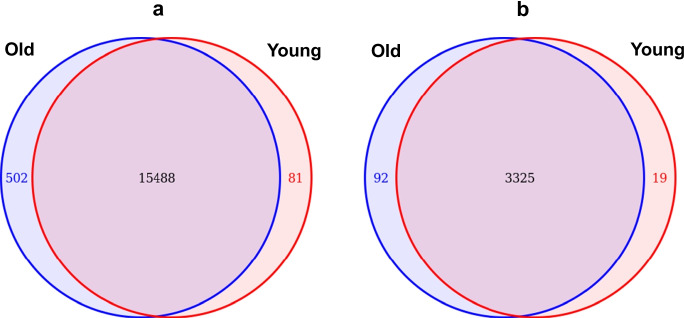
Venn diagram of all detected genes (**a**) and of the differentially expressed genes (**b**)

Out of the full set of expressed genes (16,071), 81 were found to be present exclusively in the young group and 502 were detected only in the old group. The remaining 96.4% of the genes were detected at least in one individual in both cohorts (Fig. 3a). Notably, most of the genes that were detected only in one of the cohorts were below the detection level of 0.3 CPM per gene (which corresponds to approximately 20 reads per gene) in at least 50% of the animals in the other cohort.

The differential gene expression analysis yielded 3436 genes, which were found to be significantly differentially expressed in the brains of young and old dogs (*q* value < 0.1). As the sample processing pipeline had not allowed efficient removal of blood from the brains, this list might have contained some blood-derived DEGs as well. However, when the expression levels of transcripts known to be highly expressed in red blood cells [82] were checked, no indication of major contamination was detected as the average CPM values of the three expressed genes were generally low (Table S4). Also, the pattern of relative expression levels between individual animals was inconsistent with the MDS, suggesting that at least the multidimensional scaling was not affected by blood contamination.

Interestingly, 97% (3325) of the differentially expressed genes were present in both cohorts (Fig. 3b). The observed high overlap between the 2 clusters indicates that (1) the genes that are turned on/off during adulthood are relatively rare and (2) quantitative changes in gene expression levels were mainly responsible for the clustering of animals. Importantly, the number of genes exclusively expressed in one cohort was much lower in the DEG set, as in the full set of expressed genes. This means that most genes showing different occurrences in the two groups in the total set did not reach the level of statistical significance in applied statistical models in the edgeR package. Given the high RNA quality and depth of coverage of our data, it is likely that these genes are truly expressed at a low level in both cohorts; therefore, the significance of difference cannot be reliably determined by the applied statistical methods. The most significantly differentially expressed genes were *actin-related protein 3B* (*ACTR3B*) (ENSCAFG00000004971) and *retinoic acid receptor responder 2* (*RARRES2*) (ENSCAFG00000004555) in the downregulated and upregulated sets, respectively (Fig. S3a, b).

**Fig. 4:**
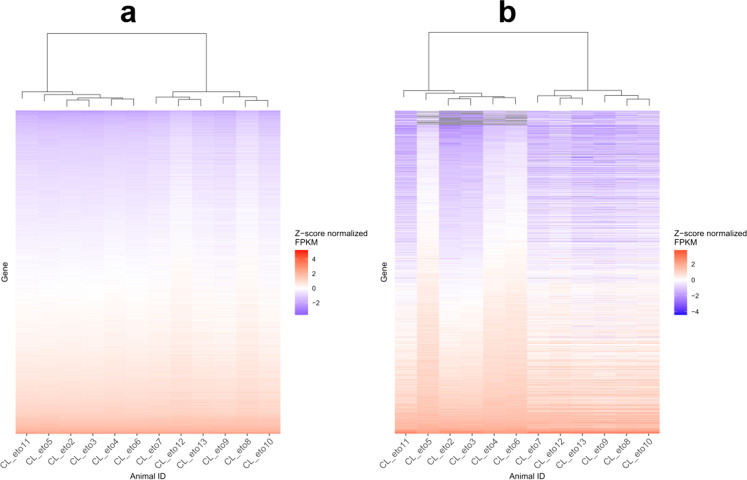
Two heatmaps showing the expression levels of the 3436 differentially expressed genes (DEGs) together with a cluster analysis of the individuals (shown on top of each heatmap) based on the DEGs. Here, the CL1_eto1 animal was excluded from the differential gene expression analysis. **a** DEGs with small (< 50%) fold change differences (2200 genes). **b** DEGs with large fold change differences (1236 genes). Genes with > 50% differences in fold change were classified as highly differentially expressed genes

Among the differentially expressed genes, the ratio of downregulated and upregulated (measured from the young to the old cohort) genes was close to 1:1, with slightly more genes being upregulated. In total, 1701 genes were downregulated from the young to the old cohort, while 1735 genes were upregulated. Fold changes ranged from 0.05 to 0.91 in the differentially expressed, downregulated genes with an average fold change of 0.73. The fold change ranged from 1.1 to 318.1 in the differentially expressed, upregulated genes (average: 2.4). Out of the 1735 upregulated genes, 339 (19.5%) showed a higher than 1.5-fold increase, while 106 (6.2%) out of the 1701 downregulated genes showed a 50% or higher decrease in expression levels. In the full set of genes, 2200 genes showed < 50% change in their expression level, while 1236 genes exhibited a change greater than that. For easier visualization, the gene expression–based clustering of the animals, which was performed based on the total set of DEGs was depicted in two different panels, representing the < 50% and > 50% fold-change DEGs separately (Fig. 4). Interestingly, CL_eto11 was grouped within the young cluster in this analysis; however, when the cluster analysis was performed on the set of DEGs derived including CL_eto1 in the analysis (Fig. S2), CL_eto11 was in the old cohort.

Altogether, 9.9% of all DEGs were at least two times more active in old dogs when compared to young dogs, i.e., their average gene expression levels estimated from RNA-Seq data were at least two times higher in the old cohort. In contrast, only 3.1% of the DEGs were at most half as active in old age compared to younger individuals. Among the upregulated genes, *CD300 H immune receptor* (*CD300H*) (ENSCAFG00000028912) (Fig. S3c) showed the greatest change in its transcript abundance with a 318.1 × fold change between the young and old cohorts. In the downregulated group, the greatest decrease in expression levels belonged to the *troponin T2, cardiac type* (*TNNT2*) gene (ENSCAFG00000010798), showing an average 25 × decrease (4% of the young animals’ average) (Fig. S3d). However, a more detailed analysis revealed that this large difference was caused by only a few young animals showing highly increased expression in the case of the *TNNT2* gene. For the *CD300H* gene, the increased expression was more generalized in older animals. However interestingly, when randomized analyses were run to assess the effect of two males present in the old cohort, *CD300H* was dropped from the DEG list in three out of five permutations, while *TNNT2* was consistent as a DEG.

**Fig. 5:**
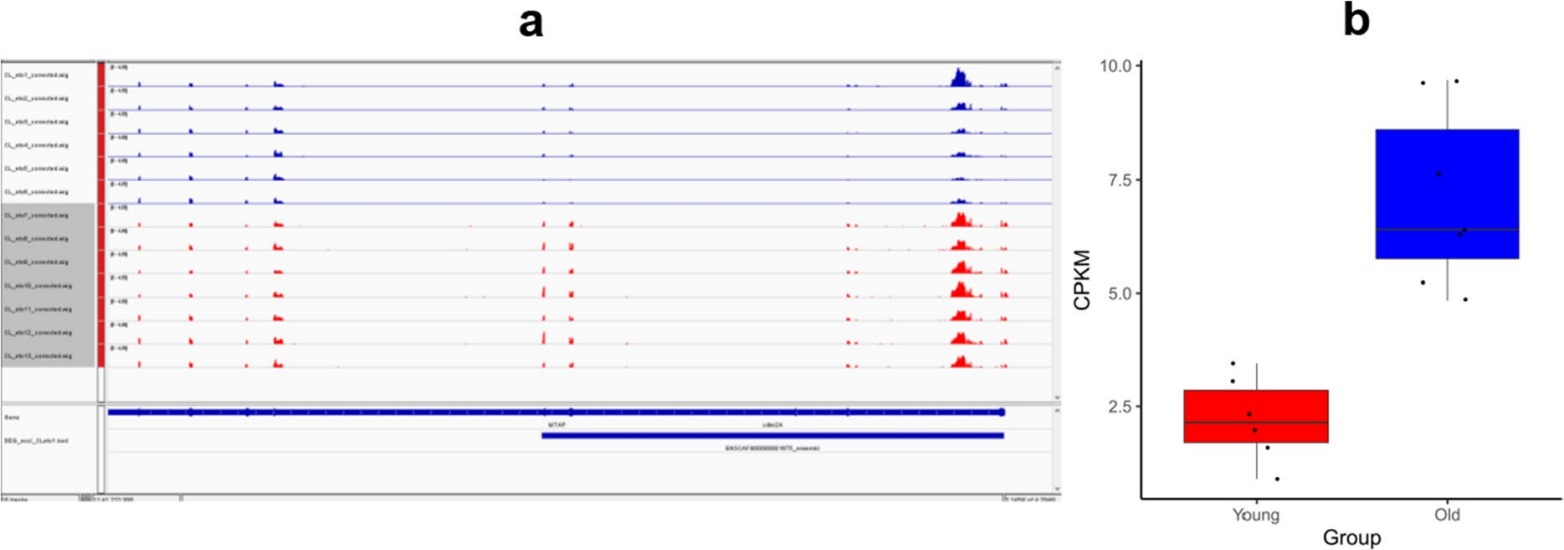
(**a**) IGV picture of the CDKN2A gene and (**b**) the distribution of the observed, corrected CPKM values shown in a boxplot. Black dots on the boxplot correspond to the individual expression levels measured in the 6 young and 7 old studied dogs

An example of a significantly differentially expressed gene (*CDKN2A*) can be seen on an IGV image in Fig. 5a. After bias correction and modeling, the same gene’s corrected expression levels (measured as *counts per million sequenced reads per kilobase gene length* or CPKM) are plotted on a boxplot (black dots correspond to individual expression levels; Fig. 5b; this figure includes CL_eto1 as well). The estimated fold change difference at this locus measured from the young to the old dogs is 3.6 × (or 3.2 with CL_eto1 included). The first young animal - CL_eto1 - is a clear outlier in the young cohort with some resemblance to the elderly dogs.

Interestingly, the list of differentially expressed genes included previously not annotated transcripts (for example, ENSCAFG00000040732 was upregulated in old animals; fold change (FC) = 5.7) and a total number of 103 long non-coding RNA genes as well. However, further analyses of the possible function of these genes were not performed within the frames of this study.

Finally, when we compared the list of differentially expressed genes with the previously reported 963 differentially expressed genes detected by microarray technology in old and young beagle dogs’ brains [66], we found 125 genes, which overlapped between the two studies (Table S5).

### Gene ontology analysis of the differentially expressed genes

**Fig. 6:**
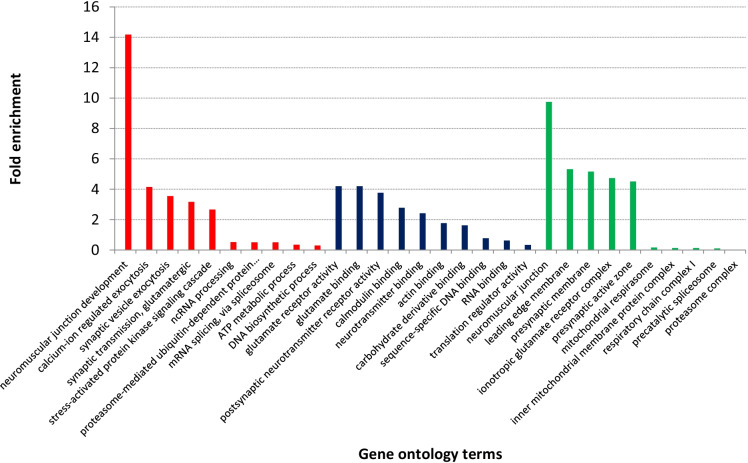
Results of the gene ontology analysis. Gene ontology terms are ordered by fold enrichment and grouped by gene ontology classes (biological process: red; cellular components: blue; molecular function: green)

A GO analysis was implemented to investigate which genetic regulatory pathways and biological mechanisms are the most overrepresented by the set of differentially expressed genes. As the samples were collected from the brains of dogs, many gene ontologies related to brain development, brain activity, etc., could be expected to be enriched, when comparing the gene set of differentially expressed genes to the whole canine transcriptome in a statistical overrepresentation test. Therefore, we defined the background gene set as the combined set of all genes that were expressed in at least one canine brain sample. Figure 6 shows the significantly enriched GO terms, colored by GO primary classes and ordered by a decreasing estimated fold enrichment (ordering was done only within class, to enhance visibility). Due to space limitations, only the five gene ontologies with the highest fold enrichment and the five with the lowest fold enrichment are plotted for each primary class. The complete list of all significantly enriched GO terms can be found in Table S6. The GO terms plotted in Fig. 6 and Table S6 include only the most specific GO terms from the gene ontology *slim* subset of the PantherDB software (v. 16.0); all other significant GO terms can be found in Supplementary Data 1.

Many of the enriched gene ontology terms were related to the nervous system. These enriched gene ontologies were related to signal transmissions, such as the following (the 2-letter abbreviation of the ontology classes is shown in parentheses): synaptic transmission, glutamatergic (biological process (BP)), neurotransmitter secretion (BP), modulation of chemical synaptic transmission (BP), regulation of membrane potential (BP), neuromuscular junction (cellular component (CC)), presynaptic active zone (CC), axon terminus (CC), signaling receptor activity (molecular function (MF)), or postsynaptic membrane (CC) and postsynaptic density (CC).

Gene ontologies related to RNA maturation were also enriched, such as ncRNA processing (BP), spliceosomal snRNP complex (CC), RNA binding (MF), or mRNA splicing, via spliceosome (BP). In addition, other, more general ontologies, such as catalytic complex (CC), can be related to RNA maturation as well.

Some genes that were highly downregulated in the old cohort were related to a diverse set of neural functions. Interestingly, several genes linked to inhibitory neurons, like *calbindin 1* (*CALB1*) (ENSCAFG00000009015), *glutamate decarboxylase 1* (*GAD1*) (ENSCAFG00000012560), and *somatostatin* (*SST*) (ENSCAFG00000013891), also showed marked downregulation in older animals (Fig. S3e–g).

Another significant enrichment in GO terms was linked to the regulation of gene expression. Genes with such GO categories included the *NPAS4* (ENSCAFG00000012711; FC ~ 0.03) (Fig. S3h) gene, which is related to the regulation of transcription and is also related to learning, short- and long-term memory, and regulation of synaptic plasticity.

Although no significant GO enrichment was found in relation to immune function, when the whole set of DEGs was analyzed, several of the genes, which were strongly and generally upregulated in the old cohort, were linked to immune response. For example, *UPK1B* (ENSCAFG00000010888; FC ~ 15.8) (Fig. S3i) is related to response to bacteria and epithelial cell differentiation; *SLC47A1* (ENSCAFG00000018195; FC ~ 17.9) (Fig. S3j) is related to xenobiotic transport; *SERPINE1* (ENSCAFG00000013909; FC ~ 10.9) (Fig. S3k) is related to chronological cell aging, to the positive regulation of interleukin-8 production and to angiogenesis; and *CCL5* (ENSCAFG00000018171; FC ~ 16.24) (Fig. S3l) is related to monocyte chemotaxis and inflammatory response or to positive regulation of T cell chemotaxis. Also, when only the set of upregulated DEGs was analyzed, two GO terms linked to immune function were found to be enriched: inflammatory response (GO:0,006,954; FE: 3.99) and activation of immune response (GO: 0,006,955; FE:2.73).

### Alternative splicing events

**Fig. 7:**
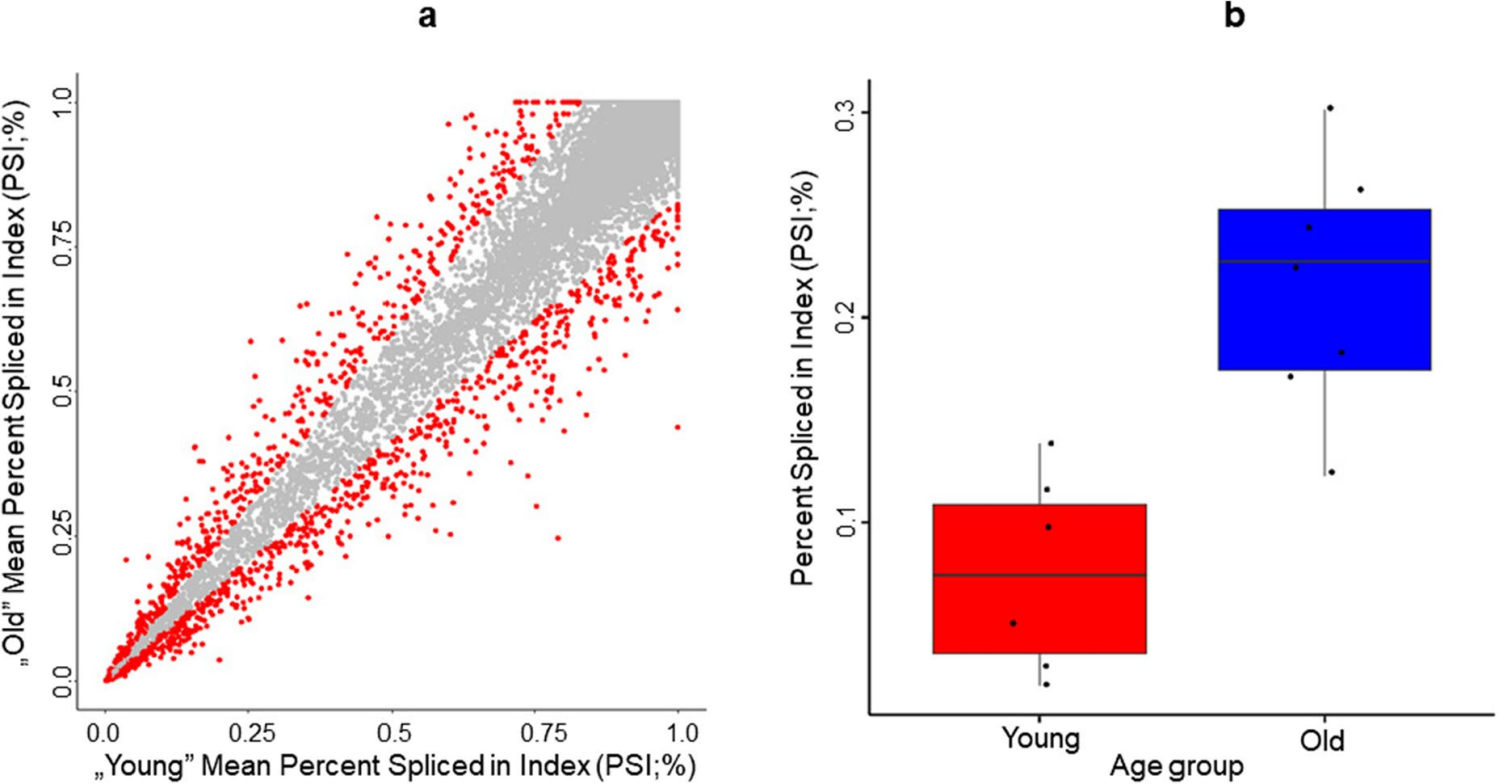
Results of the PSI analysis. **a** A scatter plot showing the estimated PSI indices in young vs. old animals. The PSI indices with > 20% difference are shown in red, while those with < 20% difference between the two cohorts are shown in gray. **b** A boxplot of PER3’s estimated PSI values with extremely divergent PSI indices between the young and old cohorts

Figure 7 shows the distribution of the estimated PSI ratios in young vs. old animals. PSI values were averaged across the young and old animals for visualization. Not surprisingly, a strong correlation was observed between the two groups (*ρ* = 0.98) in the inclusion ratio of exons. However, some exons greatly differed in their PSI estimates, indicating that their inclusion in the transcripts differed significantly between the young and old animals. A higher PSI value indicates a higher proportion of transcripts including the specific exon; therefore, the exons above the diagonal of the scatter plot correspond to the exons that were more frequently included in the transcripts in old dogs while they were spliced out from the transcripts in younger animals. The exons below the diagonal correspond to the opposite case, i.e., to exons that were more frequently spliced out from the transcripts in old animals yet retained in young dogs. The exons that had a higher than 20% difference in their average PSI estimates between the young and old dogs are highlighted in red on the scatter plot (Fig. 7a), and a boxplot is also shown as an example of one such exon (Fig. 7b). The boxplot shows the changed expression level of one of the exons of the *period circadian regulator 3* (*PER3*; ENSCAFG00000019666) gene, related to the circadian rhythm, but also RNA transcription regulation. In total, we identified 1412 exons from 1096 different genes with a difference of at least 20% in their PSI estimates between the young and old cohorts.

A gene ontology overrepresentation analysis of these 1096 genes revealed four significant gene ontologies (not counting some of their parent ontologies, which were also significant). Three of these gene ontology categories were related to protein synthesis processes (Table 3), while the fourth category contained processes linked to actin filament organization and functions based on actin filaments, like cellular movement.

**Table 3:** Gene ontology results of the 1096 genes, which included exons with very different PSI estimates in young and old animals. FDR – false discovery rate

GO biological process complete	Number of reference genes (*N* = 16,036)	Number of target genes (*N* = 1096)		Fold enrichment	FDR
		**Observed**	**Expected**		
**Actin filament–based process**	388	55	28.59	1.92	3.46E − 02
**Cellular nitrogen compound metabolic process**	2149	101	158.34	0.64	6.11E − 03
**Organonitrogen compound biosynthetic process**	888	34	65.43	0.52	3.67E − 02
**Cellular macromolecule biosynthetic process**	969	36	71.4	0.50	1.18E − 02

The reference gene list corresponds to all genes expressed in the analyzed brain tissue

### Detection of evolutionarily conserved DEGs

**Fig. 8:**
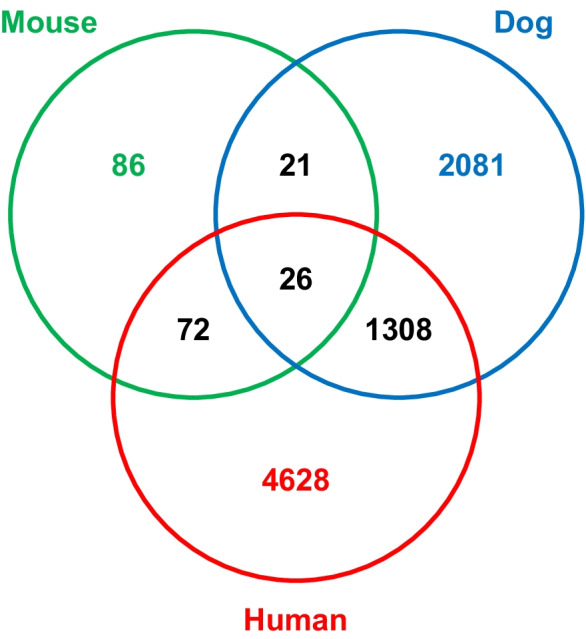
Comparison of the differentially expressed genes in dogs with those published in mice [114] and in humans [40]. In the case of the mouse and human data, only those genes were considered that had homologs in dogs

The obtained results were also compared to previously published differentially expressed age-related gene sets from human and mouse frontal cortical tissue. The two studies included in the comparison did not exactly match the current study in design and analysis pipeline; however, they seemed to be the best matching among available datasets. Therefore, the comparison included the study of Chen et al. [114], who performed an RNA-Seq experiment from the synaptosome fraction extracted from the cerebral cortex of mice, comparing the mRNA levels of young (2.5 months old; *n* = 6) and old (23 months old; *n* = 6) animals. The cohort settings, including the number of animals and their relative age (i.e., their age, after taking into consideration the species’ expected lifespan), and the analysis pipeline were very similar to our study. The human data included in the comparison was derived from the study of Dillman et al. [40], who used mRNA sequencing to investigate gene expression changes in the cerebral cortex of the frontal lobe of the human brain in a continuous age cohort (from 15 to 79 years (*n* = 56)). Here, the analysis protocol differed from ours significantly. To make the list of differentially expressed genes comparable across species, first, the Ensembl IDs were extracted for all DEGs. Then, the genes from both the human and mouse samples with homolog pairs in dogs were kept. This resulted in a list of 205 and 6034 differentially expressed genes from the mouse and human studies, respectively. A Venn diagram is shown in Fig. 8, summarizing the results of this comparison. The most prominent difference between the three studies is the number of DEGs (highest in humans, lowest in mice). This may be a result of the different sample types, as Chen et al. [114] focused on a subfraction of the cerebral cortex, namely the synaptosomes. The overlap between the three studies was low, which may be at least partly explained by the different analysis methods implemented in the three studies. The list of the 26 genes found to be differentially expressed in all studies is reported in Table S6.

### Real-time qPCR validation

The results of the RNA sequencing experiment were validated using RT-qPCR. A total of 10 differentially expressed genes were chosen from both the upregulated and downregulated list, including genes with high and moderate fold changes. Genes with fold changes smaller than 2 were not included in the validation due to the lower sensitivity of RT-qPCR relative to RNA sequencing. *GAPDH* was used as an endogenous control gene in this experiment to allow normalization of expression levels using the ΔΔCt method. Previously, *GAPDH* was checked as not being included in the list of genes, which were differentially expressed between the two age groups. We found a strong correlation between the PCR and RNA-Seq fold changes (Pearson correlation, *r* = 0.96, *p* < 0.01). Results are shown in the supplementary materials (Table S8).

## Discussion

In the current study, we used poly(A) capture mRNA sequencing to investigate the gene expression profiles of the frontal cortices of six young and seven old dogs representing various breeds. Our aim was to explore how aging affects the frontal lobe in a species with high translational relevance in dementia research. To the best of our knowledge, this is the first canine aging RNA-Seq study to investigate a range of different dog breeds, which represent the variability of natural populations of companion dogs regarding the genetic background and environmental effects. We found that in our dataset, chronological age was the most important factor to account for the observed variance in the differentially expressed genes. Three thousand four hundred thirty-six genes were either upregulated or downregulated in the old cohort compared to the young dogs. Other factors, such as sex, breed, or body size, had much smaller effects.

To our knowledge, only one study explored the age-related transcriptomic changes in the cerebral cortices (no exact region was defined) of laboratory beagle dogs (6 young and 6 old individuals) using Affymetrix GeneChip® Canine Genome Arrays [66], and no previous canine RNA-Seq study has focused on age-related transcriptional changes in the brain of dogs [35–37, 83]. Also, the high quality of the raw data in our study (an average of 74 million reads generated for each individual sample, which fulfilled the quality recommendations suggested by ENCODE (~ 30 million reads; ENCODE guidelines) by a large margin) ensures that our dataset could be a valuable addition to the already present dog transcriptomic data collection for future studies.

Out of the set of previously described canine genes (~ 20,000 genes) and transcripts (~ 45,000 transcripts), ~ 16,000 genes with ~ 38,000 known transcripts were found to be expressed in the prefrontal cortex of at least one individual in our dataset. Regarding sample size, our study included the largest number of samples (*N* = 13) in comparison to other dog RNA-Seq studies reported so far [35–37, 84]. This sample size was also slightly higher than in the study of Swanson et al. [66] on laboratory dogs; however, our sample with eight breeds and mixed-breed dogs showed a more variable genetic background. Ideally, an even greater number of dogs would have been favorable because of the genetic variance; however, the availability of donated pet dogs limited the number of included samples. Still, this sample number seemed technically appropriate to provide an acceptable power for the analysis to detect significantly differentially expressed genes in an RNA-Seq study [67, 68].

The breed heterogeneity, however, represented a less explored source of possible noise in the analysis, as no previous data was available on the variability of transcriptomes between dog breeds. Although gene expression studies conducted in mice suggested that the genetic background, e.g., in different strains, may affect only a small proportion of expressed genes [85–87], it had not been clarified whether the same applies to dog breeds. Recently, Megquier et al. [36] reported that tissue type was the single most relevant factor to contribute to the expression pattern observed in tissues derived from a group of six adult dogs representing different breeds. In accordance with these previous reports, our data indicated that the breed of the dogs had a relatively small contribution to the summarized variance of gene expression levels. Notably, the three beagle dogs in the young cohort were separated from each other in all the applied analyses (Figs. 2, S1, and S2). Altogether, these findings indicate that general gene expression patterns in certain tissues might be relatively consistent between individuals of the same species, at least on the mRNA level.

Interestingly, both the hierarchical clustering based on DEGs and the multidimensional scaling with all the 16,000 active genes identified the same animal (ID: CL_eto1) as an outlier (Figs. 2 and S2). Although the occurrence of outliers in age-related gene expression analyses was reported as a common phenomenon in both mice and human studies [88], possibly as a consequence of technical errors, there are a few plausible biological explanations for the outlier animal in our dataset. First, this dog was the oldest individual (at 4 years old) in the young cohort and belonged to a large-sized breed, namely the German shepherd dog. Since large dog breeds are known to have a shorter expected lifespan [89] and the median lifespan of this breed is 10 years [90], this dog could be considered, if not old, at least biologically older than the other (1–3-year-old) animals in the young cohort. This is also supported by the notion that the German shepherd dog showed brain gene expression patterns somewhat representing an intermediate case between the two cohorts in the hierarchical analysis of the differentially expressed genes (Fig. S2). The final list of differentially expressed genes in our analysis was derived after excluding CL_eto1 from the analysis. The exclusion of this animal resulted in a pronounced increase in the number of differentially expressed genes between the two age cohorts, indicating that despite its relatively young age (4 years), this animal had several genes showing an expression level similar to the old dogs.

Another special case was CL_eto11, a 14-year-old mixed-breed dog (which phenotypically resembled a Labrador retriever). This animal was clustered with the young cohort in the cluster analysis based on DEGs when CL_eto1 was excluded (Fig. 4). However, when the clustering based on DEGs was completed with the CL_eto1 animal included, this dog was properly positioned within the old cluster (Fig. S2). More importantly, this animal was positioned much closer to the average of the old dogs in the MDS analysis than to any of the young animals. Therefore, although CL_eto11 might had fewer of the DEG genes showing an *old* expression level, the general expression landscape of this sample still resembled the old group. The possible biological reasons for this could not be detected within the frames of this study.

Altogether, we found that the two groups of dogs in our study were clearly separated from each other based solely on their gene expression patterns, with the CL_eto1 animal being the only exception. Therefore, the overall within-group variance in both the young and old cohorts was considerably smaller than the between-group variance, supporting that age had the largest effect.

Consistent with this, we found no major effect, when we performed the differential gene expression analyses without the two male animals present in the old cohort (Table S3). Importantly, the cohort setting of this study would not allow the investigation of the presence or lack of sex-specific age-related gene expression changes. We could only state that the inclusion of two male dogs in the old cohort did not affect the final DEG list more than the presence of any of two random females. As sex-specific patterns of age-related gene expression changes have been reported in humans and mice [91], this question will require more focused investigations in the case of dogs.

### Differentially expressed genes

The final number of genes identified as DEGs after excluding CL_eto1 was 3436. Although the focused investigation of homologs of human transcripts known to be highly expressed in red blood cells [82] suggested that no major effect of residual blood contamination was present, it cannot be excluded that some of the differentially expressed genes may represent transcripts derived from blood. In the future, studies, which investigate age-related gene expression changes in dog blood, may help to clarify this question.

Interestingly, the final list of DEGs included not only mRNA transcripts. Most importantly, a total number of 103 long non-coding RNA (lncRNA) genes were found to be differentially expressed between the two age groups. It is not uncommon for lncRNAs to possess a poly(A) tail; therefore, they could be detected together with mRNAs in our dataset [92, 93]. As an increasing body of evidence suggests that lncRNAs may play an important regulatory role in cellular processes and aging as well [94, 95], our dataset could add valuable information to the possible function of these transcripts in canine brain aging. Canine lncRNAs have already been targeted in previous studies to provide annotation and insight into the possible functions of these transcripts in dogs [37, 84].

In addition to the annotated transcripts, some previously unannotated genes were also detected to be highly upregulated in old dogs in our dataset (e.g., ENSCAFG00000040732; FC ~ 5.7). The identification of these unannotated transcripts, which possibly did not show high homology with any human gene to allow annotation, could hint at species-specific changes and regulatory mechanisms during aging. Similarly, in the case of genes, the fact that robust age-related changes were detected in dogs, but not in humans, could also indicate species-specific processes, as suggested in the case of *CD300H* based on the current findings (see below).

Ninety-seven percent of all differentially expressed genes were expressed in both cohorts (Fig. 3b), indicating that age-related changes were mainly quantitative in the mRNA profile. Microarray data from other species also indicated that aging, in general, is characterized by more subtle changes in gene expression than, for example, diseases [96]. The ratio of upregulated and downregulated DEGs (measured from the young to the old cohort) was close to 1:1, with somewhat more genes (*n* = 1735) being upregulated. However, the range of the fold change was more varied: genes upregulated in the old cohort showed a greater range of fold changes, in general, than downregulated genes. Specifically, one gene (*CD300H*) in the upregulated set was up to 318 times more expressed in the old dogs, while the lowest expression of a downregulated gene was around 4% of the young cohort’s expression level. In the few cases where the expression was detected only in one cohort, a larger number of transcripts were present exclusively in the old dogs (Fig. 3b). Although it is hypothesized that global loss of heterochromatin in the genome may contribute to a general increase in gene expression with aging [97, 98], data from the human literature is controversial. For instance, Peters et al. [39] found more downregulated than upregulated genes in a gene expression study, which included blood samples from 14,983 individuals. In contrast, de Magalhães et al. [99] found somewhat more significantly upregulated genes than downregulated ones. Our results were similar to those of de Magalhães et al. [99]; while the number of upregulated genes only minimally exceeded the number of downregulated genes, it seemed that in general, the transcriptional de-repression was more pronounced than the transcriptional silencing.

It is important to note that some of the genes with an extremely high fold change between the two cohorts (i.e., those with > 100 × fold change) were expressed at a relatively low level in both cohorts when compared to the full transcriptome. Their expression was close to a zero level in the cohort showing lower expression. Therefore, although the fold changes were technically high in these genes and hence were detected to be significant by the statistical pipeline, their absolute expression levels were low in both cohorts. Also, individual investigation of some of these DEGs revealed that major individual differences arose between animals in some cases, meaning that some of these genes showed a high expression in only a few animals in either of the cohorts. Although the current analysis pipeline detected these genes as DEGs in relation to aging, their expression might have been affected by other factors on the individual level. Examples of the highly upregulated genes, which were only upregulated in a few old individuals, included the *IBSP* gene (ENSCAFG00000009558; FC ~ 13.4) (Fig. S3m) related to ossification and bone mineralization and the novel gene (ENSCAFG00000020220; FC ~ 12.7) (Fig. S3n) with gene ontologies such as acute inflammatory response or response to bacteria. Since these genes were not universally upregulated in all old samples and their baseline expression was close to 0 in most animals, they were probably related to specific circumstances of the individuals (e.g., a recent infection) and not specifically to aging. Similarly, some differentially expressed genes had an increased expression only in a few young dogs, while their expression was close to zero in other animals in most cases. Such genes included the *TNNT2* (Fig. S3c), which was found to be the most downregulated gene in the old dogs altogether, based on the applied statistical pipeline.

More specifically, in the case of *TNNT2*, individual-level analyses revealed that the group difference was a result of two young dogs showing increased expression (Fig. S3c). Consequently, these extreme fold change values were again likely resulting from specific individual circumstances apart from chronological age. Nevertheless, a connection between *TNNT2* and aging might exist; a deletion mutation resulted in premature death in knock-in mice due to cardiac dysfunction [100].

In the case of the second most downregulated gene, *endothelin-converting enzyme like 1* (*ECEL1*) (ENSCAFG00000011211), a more general difference was observed among the two age groups (Fig. S3o). *ECEL1* plays an important role in the development of the central nervous system [101]; thus, it belongs to one of the enriched GO categories found in the current study.

Contrasting the individual-specific expression pattern of *TNNT2*, the most upregulated gene, *CD300H*, showed a robust increase in all old dogs (and in CL_eto1; Fig. S3c). Human *CD300H* was recently identified as a new member of the CD300 immunoreceptor family and was hypothesized to play an important role in innate immunity [102]. For this gene, no mouse homolog has been reported, and a common SNP was found to result in a loss of its expression in humans. Therefore, this gene may represent an example of genes, which play a non-conserved, species-specific correlation with chronological age. Whether there is a functional role of *CD300H* in canine brain aging should be investigated in future studies. Such unique differences in the regulation of aging may be especially relevant in translational studies. Further investigations are also demanded because, intriguingly, CD300H was missing from the DEG list in some cases, when we performed the analysis in random permutations of excluding two old animals or excluding the two males. This indicates that the detected pattern of age-related expression of *CD300H* could be majorly affected by individual differences.

The most significantly differentially expressed genes were *ACTR3B* and *RARRES2* in our study (Fig. S3a and b). One previous human study identified *ACTR3B* as an important hub in the age-related gene expression network changes in the human frontal cortex [103]. The other three genes mentioned by Hu et al. [103] (*CAMSAP1*, *PPP3CB*, *GNG1*) were also present in our dataset with a relatively low *p* value and FDR rate. In the case of the second most significant gene in our study, *RARRES2*, the low expression of its homolog was found to be a characteristic of the astrocyte fraction in the mouse brain [104] and it was associated with obesity and metabolic disease in humans [105].

### Splicing

Following the identification of differentially expressed individual genes, age-related alterations in the splicing landscape of the transcriptome were investigated. One thousand ninety-six genes were found to show age-related variance in the mRNA inclusion of at least one exon when a 20% threshold was applied for the detected difference between the two cohorts. This indicates that changes in alternative splicing mechanisms are typical in dogs as well as in humans. The ratio of skipped/included exons in relation to age was relatively balanced (Fig. 7), indicating that fine-tuned regulatory effects rather than a generally reduced function of, e.g., exon skipping are responsible for the overall changes. A more detailed analysis of the splicing alterations was out of the scope of this study; however, subsequent investigations are yet to be performed on our dataset in the future.

### Gene ontology

To gain a more comprehensive view of the relevance and function of differentially expressed or differently spliced genes, gene ontology analyses were performed for both lists of genes (i.e., differentially expressed and altered splicing pattern). The gene ontology analysis revealed several enriched GO terms, which were connected to genetic regulatory mechanisms previously described in human aging.

Most importantly, we found several enriched GO terms linked to neural development (Fig. 6). Previous research found similar enrichment for this type of GO terms in human brain aging, in contrast to results from mice [106]. The same authors found several genes, which were downregulated only in humans and primates in relation to aging, while in mice, mainly upregulation was detected. Most of these genes were linked to specific neuron types, e.g., inhibitory neurons. In our set of differentially expressed genes, we found several examples of downregulated genes, which corresponded with the human data. For example, *CALB1*, *GAD1*, and *SST* (Fig. S3e–g), which are markers of inhibitory neurons, were all downregulated in the dog dataset. Altogether, this indicates that the molecular patterns of brain aging in canines might be more similar to those in primates than those in rodents.

We also found that genes related to RNA expression and maturation were overrepresented in the differentially expressed gene set, such as the gene ontologies of “ncRNA processing,” “mRNA splicing via spliceosome,” or “translation regulation activity” (Fig. 6). This finding is concordant with both previous reports from humans and model organisms [107–111] and with the reported splicing alterations in the current dataset. The age-related changes in genes linked to the spliceosome can contribute to the changes observed in the alternative splicing landscape. This also supports the hypothesis that regulation of gene expression could represent evolutionarily conserved, central aging mechanisms. It was previously reported, for example, that several genes experimentally linked to longevity in *Caenorhabditis elegans* played roles in the regulation of gene expression and were also conserved between worms and humans [108]. In addition, fine-tuning of the regulation of gene expression may also play a pivotal role in extreme longevity and determining the aging course (e.g., healthy vs. pathological aging) as was independently hypothesized by both Yanai et al. (2017) and Jónás et al. [20]. The current findings of the enriched representation of gene expression regulating genes in our DEG set could provide further support for this hypothesis. Although the currently available data is insufficient to draw definite conclusions regarding the exact role of gene expression regulation in longevity and healthy aging, the findings may inspire additional investigations in this direction.

In addition, many of the genes that were highly upregulated in the majority of the old animals were related to immune response. This finding was concordant with previous reports about the altered function of the immune system in aged beagle dogs [112]. Additionally, a similar, age-related enrichment of immune response–linked genes was found in humans’ blood or lymphoblastoid cell lines derived from a large cohort of people [39, 41, 113]. Interestingly, an apparent enrichment of immune function–linked genes was not reported by Dillman et al. [40], who applied co-expression network analysis to investigate the age-related transcriptomic changes in human brain tissue. However, a meta-analytic study, which compared microarray data from humans, primates, and rodents, also detected several immune system function–linked genes to be consistently differentially expressed in relation to aging [99]. Therefore, although no enrichment of these GO functions was detected, the fact that some genes with extremely high changes were linked to the immune response could still hint at the role of immune-mediated processes in the brain aging of dogs.

Altogether, the GO analyses of both gene sets (DEG and splicing) yielded interesting observations, which could support the applicability of dogs as natural models of human aging with good translatability. Further discussion of these findings is not possible at this stage, as no information is available on the direction in which the genes connected to these gene ontologies affect the related pathways (or any other significant GO from Fig. 6, Table S6, and Table 3). Also, in the case of the alternative splicing changes, the relative abundance of the different transcripts is another crucial missing information (although it could be probably estimated by combining information from the PSI estimates and the estimated relative gene abundance from the RNA-Seq data, e.g., see Fig. 7a).

### Comparison with other mammalian literature data

One previous study reported high-throughput transcriptomic analysis in relation to brain aging in dogs, using microarray technology [66]. They reported 963 transcripts to be differentially expressed between young and old beagles. When we compared our findings with their findings, 125 differentially expressed genes overlapped. This relatively small overlap between two datasets representing similar sample types and cohort settings could result from several factors, such as different expression analysis methods, different brain regions, and an additional effect of genetic variability in our dataset. The 125 genes, which overlapped between the two studies, however, could hint at important brain aging mechanisms present in dogs.

To search for evolutionarily conserved genes and genetic regulatory mechanisms affecting brain aging in dogs, we compared our results with previous mouse and human RNA-Seq studies. We chose studies from the available literature based on how their design/analysis pipeline matched the current study. Although no perfect match was found regarding the cohort composition, targeted sample type, or analysis pipeline, the studies of Chen et al. [114] and Dillman et al. [40] seemed appropriate to provide insight into the possibly conserved genes and genetic regulatory networks underlying brain aging in the three species. The main factors that limited comparability were the following: differences between cohort settings, biological sample materials, applied sequencing method, and analysis pipeline. The analysis pipeline applied by Chen et al. [114] was concordant with our pipeline (in fact, it was an earlier version of it). Also, they analyzed six young and six old mice in their study, with an average of ~ 61 million reads per sample. Therefore, both the group sizes and the generated amount of sequence data were very similar to ours (although our depth of coverage is somewhat higher with an average of ~ 72 million aligned reads, especially if we consider the slightly shorter dog reference genome, when compared to the mouse genome). However, in contrast to Dillman et al. [40] and our study, Chen et al. [114] investigated the synaptosome subtraction of the cerebral cortical tissue and used total RNA sequencing. The data analysis pipeline of Dillman et al. [40] differed from ours and that of Chen et al. [114] significantly, as they applied a whole-genome co-expression network analysis approach. Their cohort setting was also different, as the age of the sample donors in this study ranged from 15 to 79 (*n* = 56), resulting in a continuous age setting, in contrast to the large age gap between the young and old cohorts in the mouse and dog datasets. These factors combined meant that the results were not directly comparable; however, they may give insight into the most strongly conserved differentially expressed genes.

Chen et al. [114] identified 6902 differentially expressed genes, 260 of which were previously annotated on the mouse genome, while Dillman et al. [40] reported 7321 differentially expressed human genes. This large difference between the two papers was possibly a result of the different sample types (whole cerebral cortical tissue vs. synaptosome fraction) and the different cohort settings (polarized vs. continuous), both aspects which suggest a larger expected number of DEGs for the study of Dillman et al. [40]. In addition, since samples from 15–20-year-old humans were also included in the study of Dillman et al. [40], their results most likely include genes that are required for brain development prior to adulthood. The exact determination of such genes was not possible either in our dataset or in the mouse study [114], due to the study design. Altogether, with the polarized cohort setting and analysis of whole cerebral cortical tissue, our dataset was technically expected to show a midline number of DEGs in relation to the other two studies.

The 26 genes that were shared between the three datasets (Table S7) included candidates with various possible links to aging, including regulation of DNA methylation (e.g., *CEBPA*), modulation of microglial function (e.g., *CX3CL1*), reduction of oxidative stress (e.g., *LANCL1*), and contribution to develop predisposition for Alzheimer’s disease (e.g., *SORCS1*).

It is noteworthy that none of the genes overlapped between datasets when we compared the evolutionarily conversed list (Table S7) and the list of genes overlapping between the beagle microarray study [66] and our study (Table S5).

However, as stated above, several factors limited the exact comparability of the mentioned studies; therefore, the current set of identified conserved genes may be revised by including further datasets in a meta-analytic study.

### Limitations

As the availability of samples for this study depended on the randomized pattern of donation made by dog owners, the cohort constitution could not be ideally optimized for every factor. Consequently, the average body size of the dogs in the old cohort was larger (21.4 ± 6.3 kg) when compared to the young cohort (13.3 ± 5.4 kg). In addition, only two male dogs were included in the old cohort, while all other animals were females in the study. Therefore, it cannot be excluded that either the weight or sex of animals affected the results. However, the weight seemed unlikely to have a major impact on the separation of the two cohorts, as within each age cluster, the dogs with different weights seemed to be randomly distributed, based on both the multidimensional scaling plot (Fig. 2) and the cluster analysis of the differentially expressed genes (Fig. 4). Also, the CL_eto11 animal was clustered together with the young dogs (despite being 14 years old) in the cluster analysis based on DEGs derived after CL_eto1 had been excluded. As this animal was among the heavier dogs in the old group (weighting 25.4 kg), its incorrect clustering could also support the notion that the average mass difference between the two cohorts did not fundamentally impact the results of the analysis regarding the separation of the two age groups. Regarding sex, any possible sex-specific differences in the aging pattern could not be detected in our dataset as the results mainly refer to female dogs in general.

## Conclusions

This study represents the most comprehensive dog brain aging transcriptomic analysis published so far. The sample size of 13 animals and the variable genetic background of these dogs enhanced the power of the analysis to detect age-related gene expression alterations, which are universal among dogs. Furthermore, the quality of the raw data generated within the frames of this study was exceptionally good. The expanding collection of good-quality canine transcriptomic data available to scientists can become a valuable resource for further meta-analytic investigations to address different questions more deeply, e.g., about the evolution of dog breeds and phenotypes.

The main findings of the current study contribute to the validation of dogs as natural models of human neural aging. The list of genes found to be differentially expressed between young and old dogs included several genes linked to neural function, immune function, and protein synthesis, similar to humans. Most importantly, many of the neural function–linked differentially expressed genes showed an expression pattern distinct from rodents, indicating that genetic regulatory changes in canine brain aging are more analogous to those in human brain aging than those in rodents. However, we also identified highly differentially expressed genes in dogs, which did not have any known human counterparts (i.e., they were not annotated) or were not reported to show age-related changes in humans. Also, the relatively small overlap in differentially expressed genes between our dataset and a best-fitting human or mouse dataset indicates that drawing conclusions about the conserved genetic mechanisms of aging will require further investigation. Altogether, our study is the first to provide detailed insight into the age-related transcriptomic differences in dogs’ frontal cortical brain area. Our dataset and our findings can support both the canine transcriptomic research in general and the dog–human translational research.

## Supplementary Information

Below is the link to the electronic supplementary material.

Supplementary file1 (PDF 339 KB)

Supplementary file2 (PDF 63 KB)

Supplementary file3 (PDF 136 KB)

Supplementary file4 (PDF 755 KB)

Supplementary file5 (DOCX 40 KB)

Supplementary file6 (XLSX 26 KB)

## Data Availability

The raw sequence files analyzed in this study are publicly available in fastq file format at the [American] National Center for Biotechnology Information’s Sequence Read Archive under the following BioProject ID: PRJNA815057.

## Abbreviations

ACTR3B: Actin-related protein 3B
AD: Alzheimer’s disease
BAM: Binary version of SAM
BP: Biological process (as GO category)
CALB1: Calbindin 1
CAMSAP1: Calmodulin-regulated spectrin-associated protein 1
CC: Cellular component (as GO category)
CCD: Canine cognitive dysfunction
CCL5: C-C motif chemokine ligand 5
CD300H: CD300 H immune receptor
CDKN2A: Cyclin-dependent kinase inhibitor 2A
CEBPA: CCAAT enhancer–binding protein alpha
CPM: Counts per million (of sequenced reads)
CX3CL1: C-X3-C motif chemokine ligand 1
DEG: Differentially expressed gene
ECEL1: Endothelin-converting enzyme like 1
ER: Exclusion rate (of exons)
GAD1: Glutamate decarboxylase 1
GAPDH: Glyceraldehyde 3-phosphate dehydrogenase
GNG1: Guanine nucleotide–binding protein, gamma subunit
GO: Gene ontology
IBSP: Integrin-binding sialoprotein
IGF1: Insulin-like growth factor 1
IGV: Integrative Genomics Viewer
IR: Inclusion rate (of exons)
LANCL1: Lanthionine synthase C–like protein 1
lncRNA: Long non-coding RNA
MDS: Multidimensional scaling
MF: Molecular function (as GO category)
NPAS4: Neuronal PAS domain protein 4
PER3: Period circadian regulator 3
PPP3CB: Protein phosphatase 3 catalytic subunit beta
PSI: Percent spliced in index
RARRES2: Retinoic acid receptor responder 2
RIN: RNA integrity number
RT-qPCR: Real-time quantitative polymerase chain reaction
SAM: Sequence Alignment Map
SERPINE1 (PAI-1): Plasminogen activator inhibitor 1
SLC47A1: Solute carrier family 47 member 1
SORCS1: Sortilin-related VPS10 domain-containing receptor 1
SST: Somatostatin
TNNT2: Troponin T2, cardiac type
ULT: Ultra-low temperature
UPK1B: Uroplakin 1B

## Code availability

In-house scripts were written to estimate PSI indices. These scripts are available from the authors upon request.
