# Supplementary material for: Poly(A) RNA sequencing reveals age-related differences in the prefrontal cortex of dogs
Source: GeroScience. 2022 Mar 14;44(3):1269–93. doi: 10.1007/s11357-022-00533-3 (PMC9213612; doi:10.1007/s11357-022-00533-3)
Supplement: Supplementary file 1 — Supplementary file1 (PDF 339 KB) [file 11357_2022_533_MOESM1_ESM.pdf]

Supplementary document

Poly(A) RNA sequencing reveals age-related differences in the prefrontal cortex of dogs

Authors: Sára Sándor[1*], Dávid Jónás[1*], Kitti Tátrai[1,2], Kálmán Czeibert[1], Eniko Kubinyi[1]

[1]Department of Ethology, ELTE Eötvös Loránd University, 1/c Pázmány Péter sétány, Budapest, 1117, Hungary
[2]Department of Genetics, ELTE Eötvös Loránd University, 1/c Pázmány Péter sétány, Budapest, 1117, Hungary

[*]: these authors contributed equally to this work

Corresponding author: Sára Sándor
E-mail: sandorsara@gmail.com

Supplementary information

Multidimensional scaling

Multidimensional scaling was performed with the biological coefficient of variation instead of the log2 fold change values to measure distances between the animals. For this purpose, the top 500 most differentially expressed genes were considered. The biological coefficient of variation is an estimator of the unknown, true variance with which the gene expression varies between biological replicates and within a condition. For further details, see the User's Guide of the edgeR R package, available on Bioconductor (http://bioconductor.org/packages/release/bioc/html/edgeR.html; accessed: 2020/12/01).

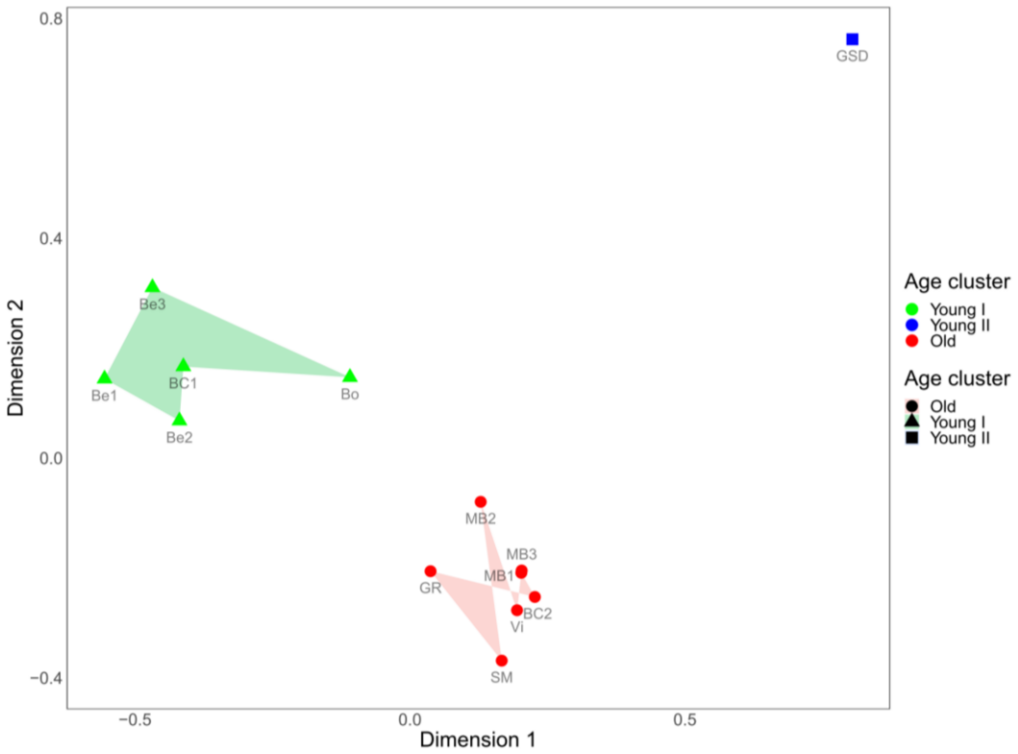

Figure s1: Multidimensional scaling of the CPM (counts per million reads) values of the samples, with distances between the individuals representing the biological coefficient of variation. The coloring of the groups is according to the visibly distinguishable clusters. Young II is an outlier young dog, a 4-year-old German shepherd dog.

Exclusion of the extreme outlier (CL_eto1)

The implemented multidimensional scaling analyses provided sufficient support for the exclusion of CL_eto1 from the differential gene expression analysis, but we also wanted to see how the number of differentially expressed genes (DEGs) changes with the exclusion of CL_eto1 compared to the analysis when all young individuals are included as well as compared to analyses when random young animals are excluded. The obtained results can be seen in **Table s2**.

Table s2: The number of differentially expressed genes (DEGs) in 4 different analyses.

Analysis	Number of DEGs	Number of down-regulated genes	Number of up-regulated genes
All individuals	1152	634	518
Excluding CL_eto1	3436	1701	1735
Excluding CL_eto3	878	477	401
Excluding CL_eto5	668	388	280

This analysis provides further support for the exclusion of CL_eto1. In short, the exclusion of CL_eto1 increases the number of differentially expressed genes between the 2 age cohorts by 3-fold, while the exclusion of other, randomly selected young animals decreases the number of DEGs by ~1/3. We conclude is that CL_eto1 introduces bias to the analysis due to its intermediate gene expression profile between the 2 age cohorts, while the other 2 individuals include useful information, which – if removed – significantly decreases the number of DEGs. This suggests that the inclusion of CL_eto1 in the analysis prevents the detection of many, otherwise differentially expressed genes between the age groups.

It must be noted that presumably, most of the ~2300 DEGs detected after the exclusion of CL_eto1 mainly show a small difference between the 2 age cohorts, which is small enough to be masked by the bias introduced with CL_eto1 in the primary analysis (i.e., in which all animals were included).

Differential gene expression analysis including CL_eto1

A separate differential gene expression analysis was implemented, which included CL_eto1 in the young cohort. This analysis resulted in 1152 differentially expressed genes (**Figure s2**), with 634 downregulated and 518 upregulated genes. In total, 102 downregulated and 99 upregulated genes showed a more than the 2-fold increase between the young and old groups. The fold change ranged from 0.02 to 0.89 in the downregulated genes (mean FC: 0.66), and it was between 1.1 and 14.6 (mean: 1.8) in the upregulated genes.
The hierarchical clustering of the animals based on the differentially expressed genes showed a similar picture to the multidimensional scaling: the young and old individuals were separated, except for CL_eto1, which was put approximately halfway between the 2 age clusters, somewhat closer to the old cohort. Age was the largest effect influencing the cluster analysis.

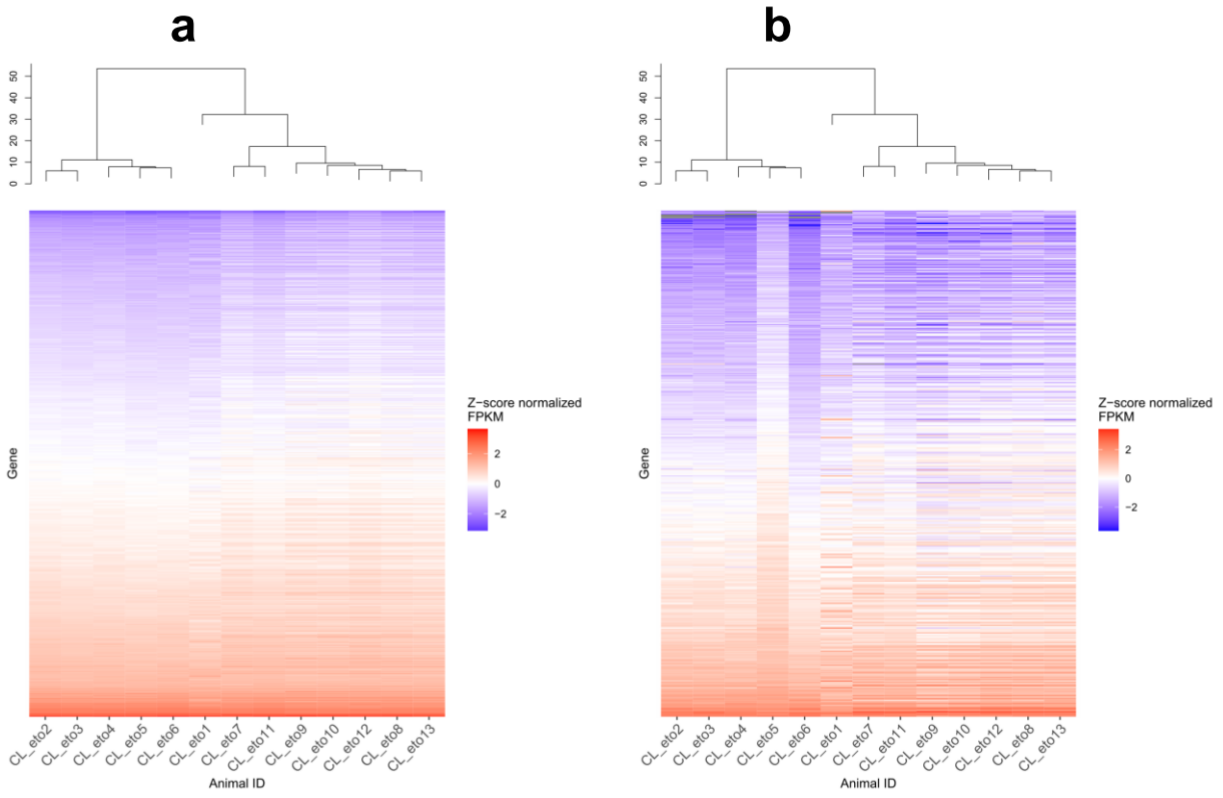

Figure s2: Two heatmaps showing the expression levels of the 1152 differentially expressed genes (DEGs) together with a cluster analysis of the individuals (shown on top of each heatmap) based on the DEGs. A: DEGs with small (< 50%) fold-change differences (641 genes). B: DEGs with large fold-change differences (511 genes). Genes with > 50% differences in fold-change were classified as highly differentially expressed genes.

Note: for the cluster analysis, all 1152 differentially expressed genes were used.
