# Supplementary material for: Poly(A) RNA sequencing reveals age-related differences in the prefrontal cortex of dogs
Source: GeroScience. 2022 Mar 14;44(3):1269–93. doi: 10.1007/s11357-022-00533-3 (PMC9213612; doi:10.1007/s11357-022-00533-3)
Supplement: Supplementary file 2 — Supplementary file2 (PDF 63 KB) [file 11357_2022_533_MOESM2_ESM.pdf]

Figure S1

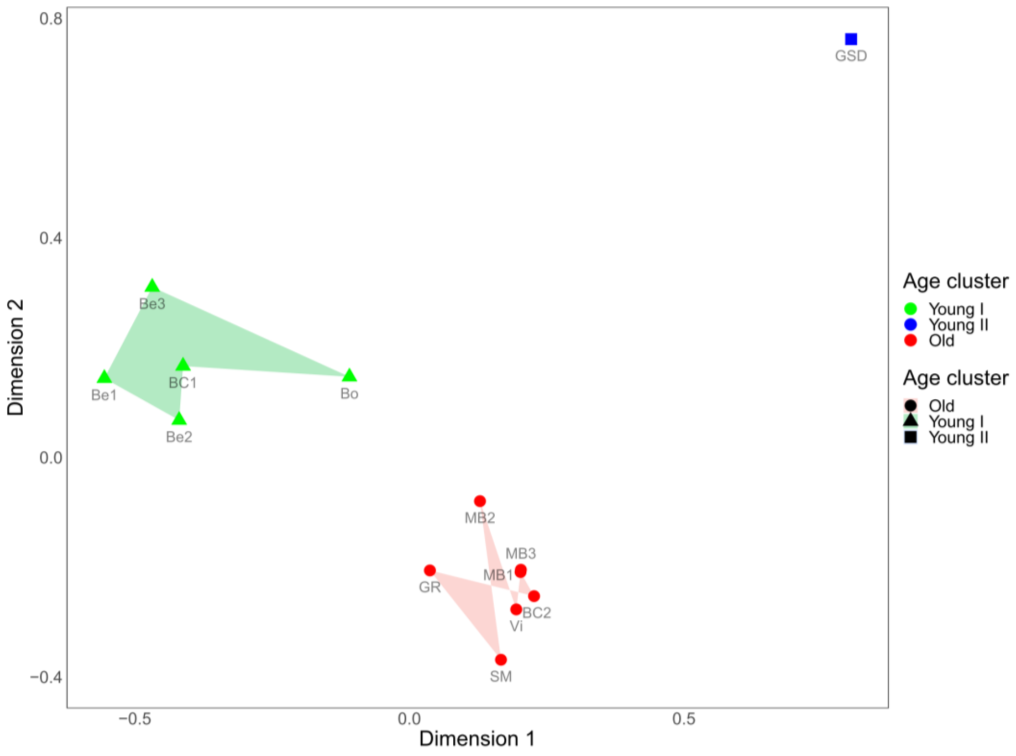

Figure s1: Multidimensional scaling of the CPM (counts per million reads) values of the samples, with distances between the individuals representing the biological coefficient of variation. Coloring of the groups is according to the visibly distinguishable clusters. Young II is an outlier young dog, a 4-year-old German shepherd dog.
