# Supplementary material for: Poly(A) RNA sequencing reveals age-related differences in the prefrontal cortex of dogs
Source: GeroScience. 2022 Mar 14;44(3):1269–93. doi: 10.1007/s11357-022-00533-3 (PMC9213612; doi:10.1007/s11357-022-00533-3)
Supplement: Supplementary file 3 — Supplementary file3 (PDF 136 KB) [file 11357_2022_533_MOESM3_ESM.pdf]

Figure S2

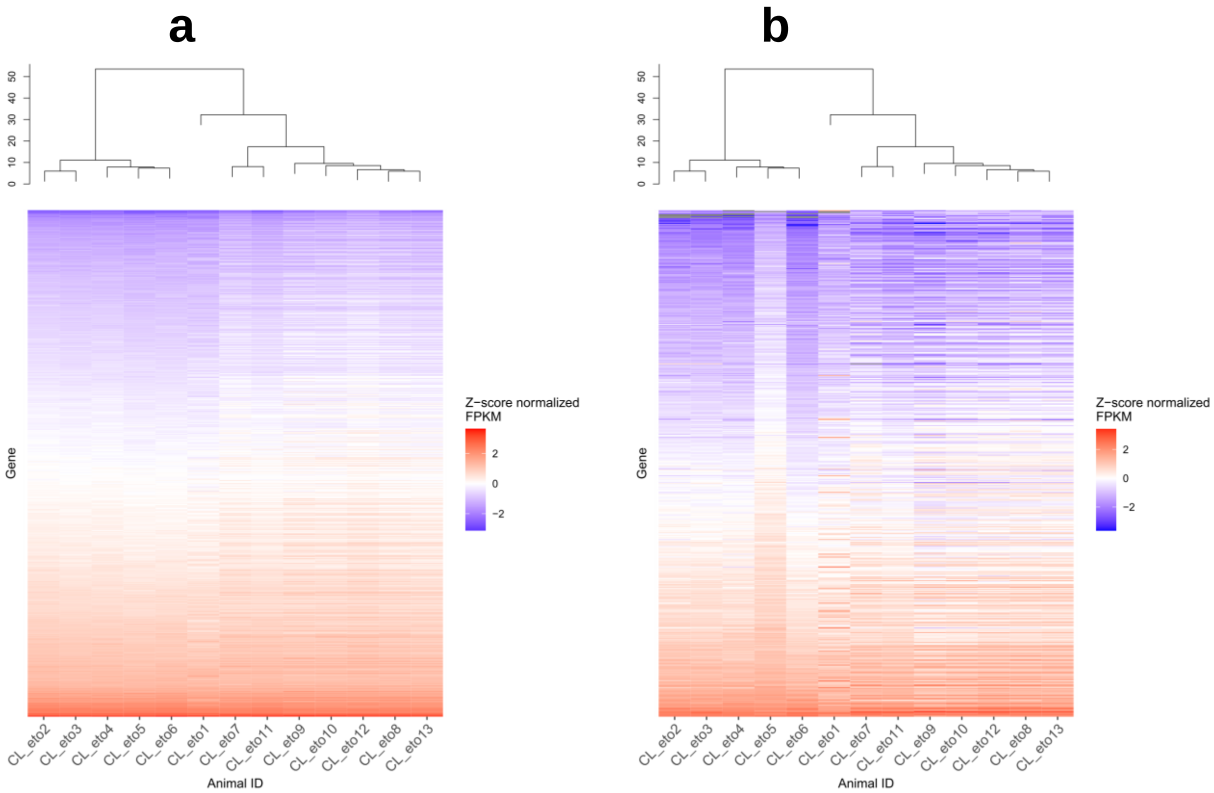

Figure s2: Two heatmaps showing the expression levels of the 1152 differentially expressed genes (DEGs) together with a cluster analysis of the individuals (shown on top of each heatmap) based on the DEGs. Here, the CL_eto1 animal was included in the differential gene expression analysis. **(a)** DEGs with small (< 50%) fold-change differences (641 genes). **(b)** DEGs with large fold-change differences (511 genes). Genes with > 50% differences in fold-change were classified as highly differentially expressed genes. Note: for the cluster analysis, all 1152 differentially expressed genes were used.
