# Supplementary material for: Poly(A) RNA sequencing reveals age-related differences in the prefrontal cortex of dogs
Source: GeroScience. 2022 Mar 14;44(3):1269–93. doi: 10.1007/s11357-022-00533-3 (PMC9213612; doi:10.1007/s11357-022-00533-3)

Figure s3

Figure s3: Expression signature for each gene mentioned in detail in the main text. The IGV 2.8.13. software was used for visualization.

a (ACTR3B)

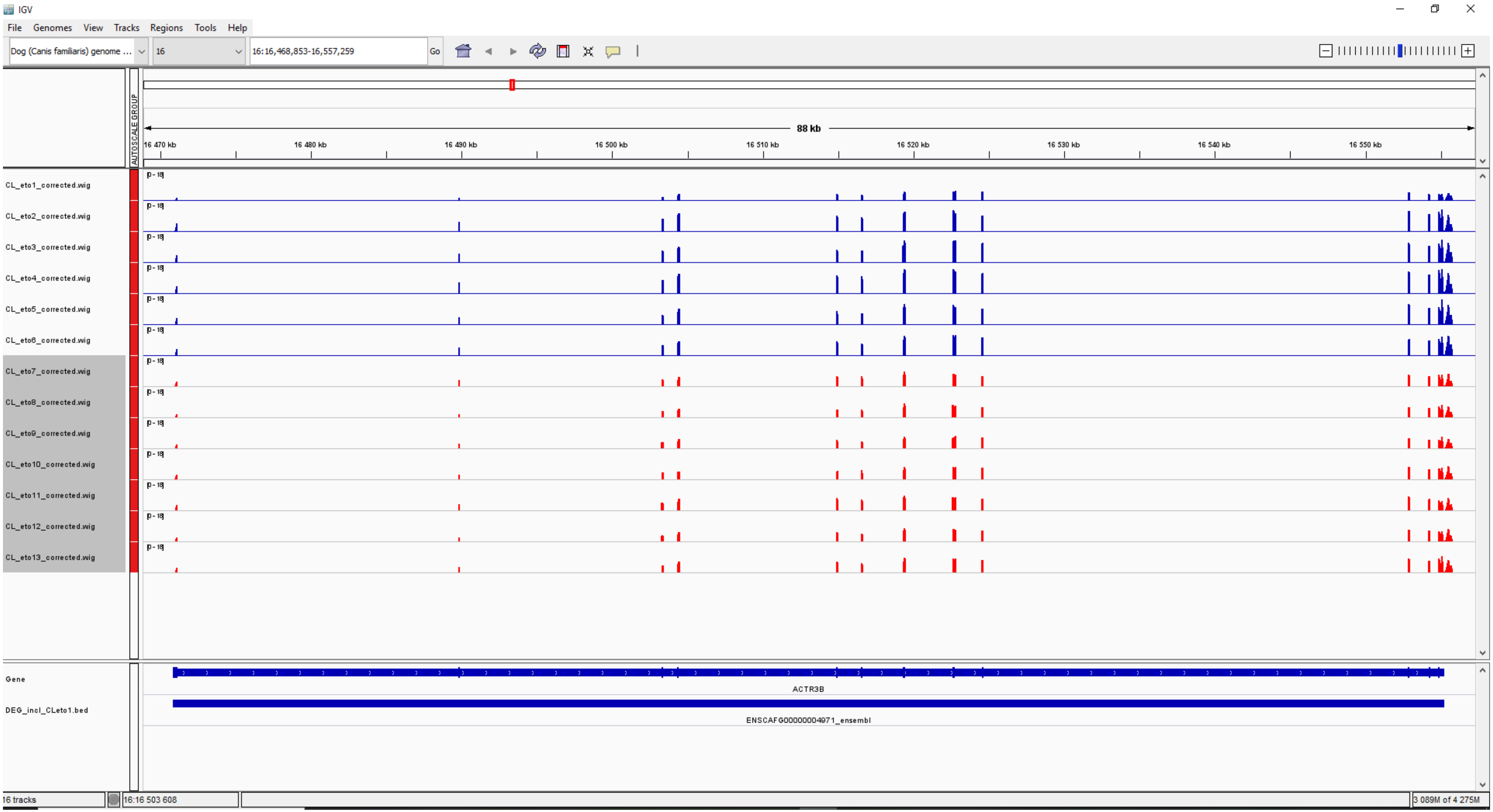

Figure s3

Figure s3: Expression signature for each gene mentioned in detail in the main text. The IGV 2.8.13. software was used for visualization.

b (RARRES2)

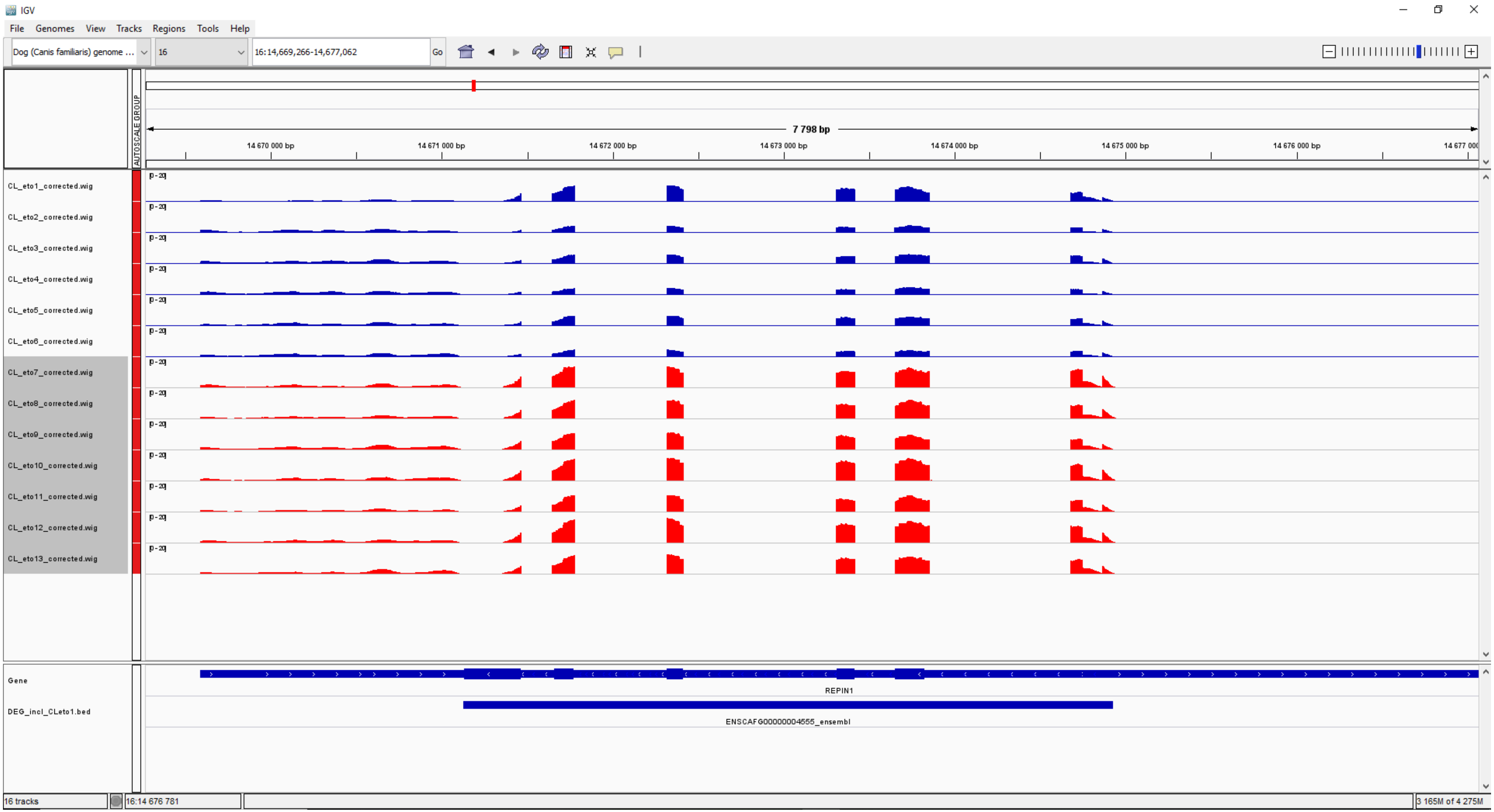

Figure s3

Figure s3: Expression signature for each gene mentioned in detail in the main text. The IGV 2.8.13. software was used for visualization.

c (CD300H)

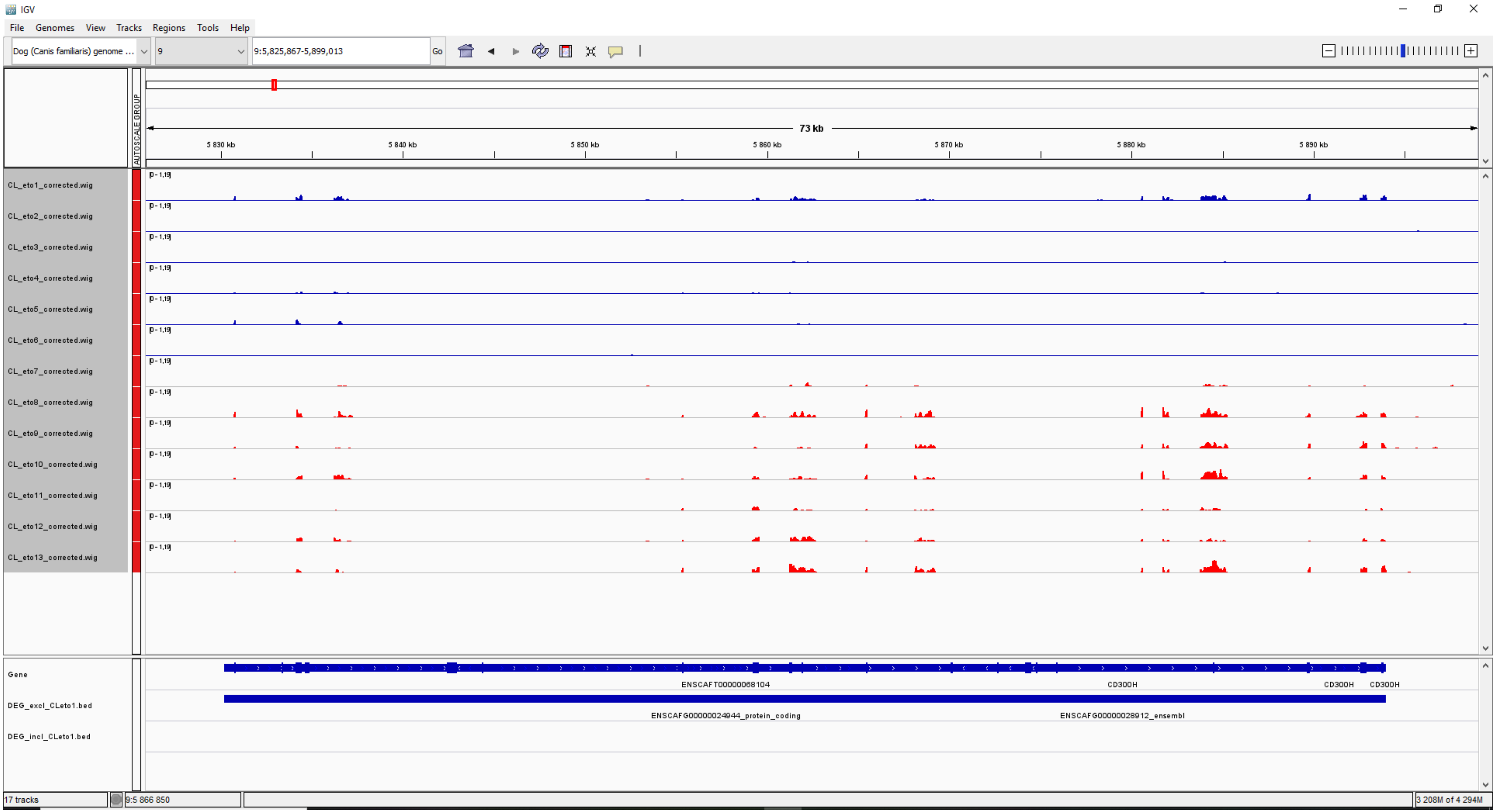

Figure s3

Figure s3: Expression signature for each gene mentioned in detail in the main text. The IGV 2.8.13. software was used for visualization.

d (TNNT2)

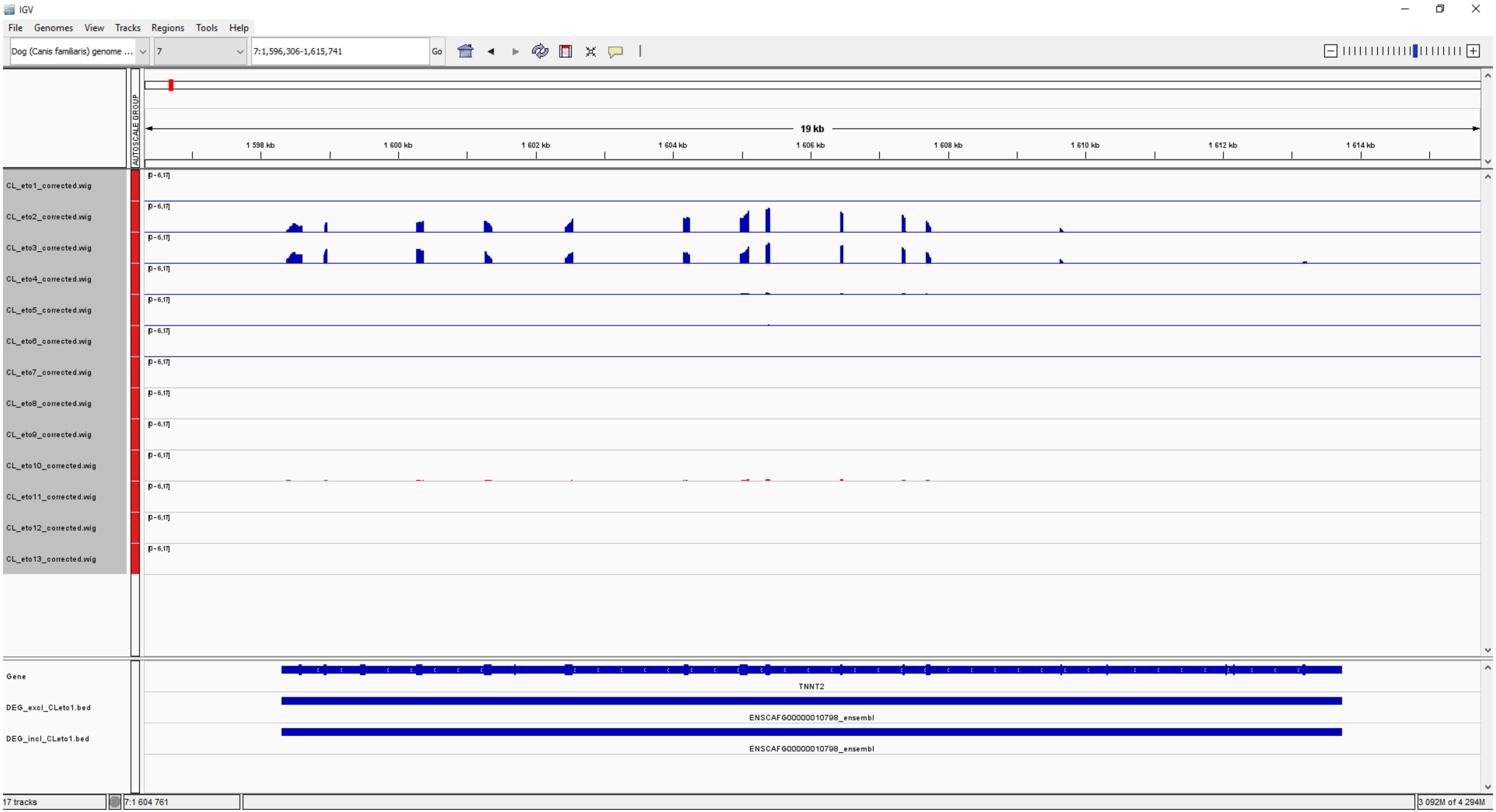

Figure s3

Figure s3: Expression signature for each gene mentioned in detail in the main text. The IGV 2.8.13. software was used for visualization.

e (CALB1)

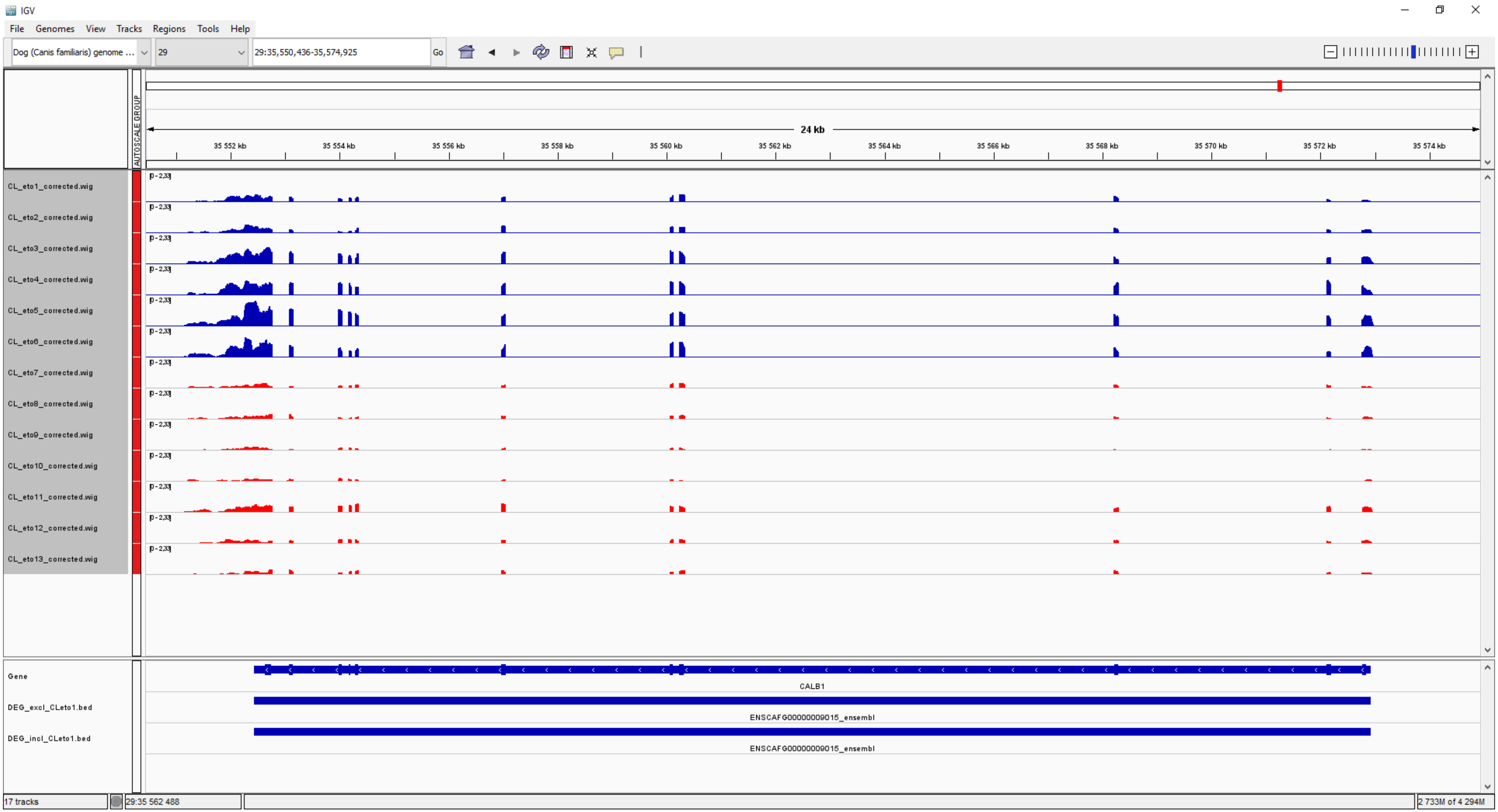

Figure s3

Figure s3: Expression signature for each gene mentioned in detail in the main text. The IGV 2.8.13. software was used for visualization.

f (GAD1)

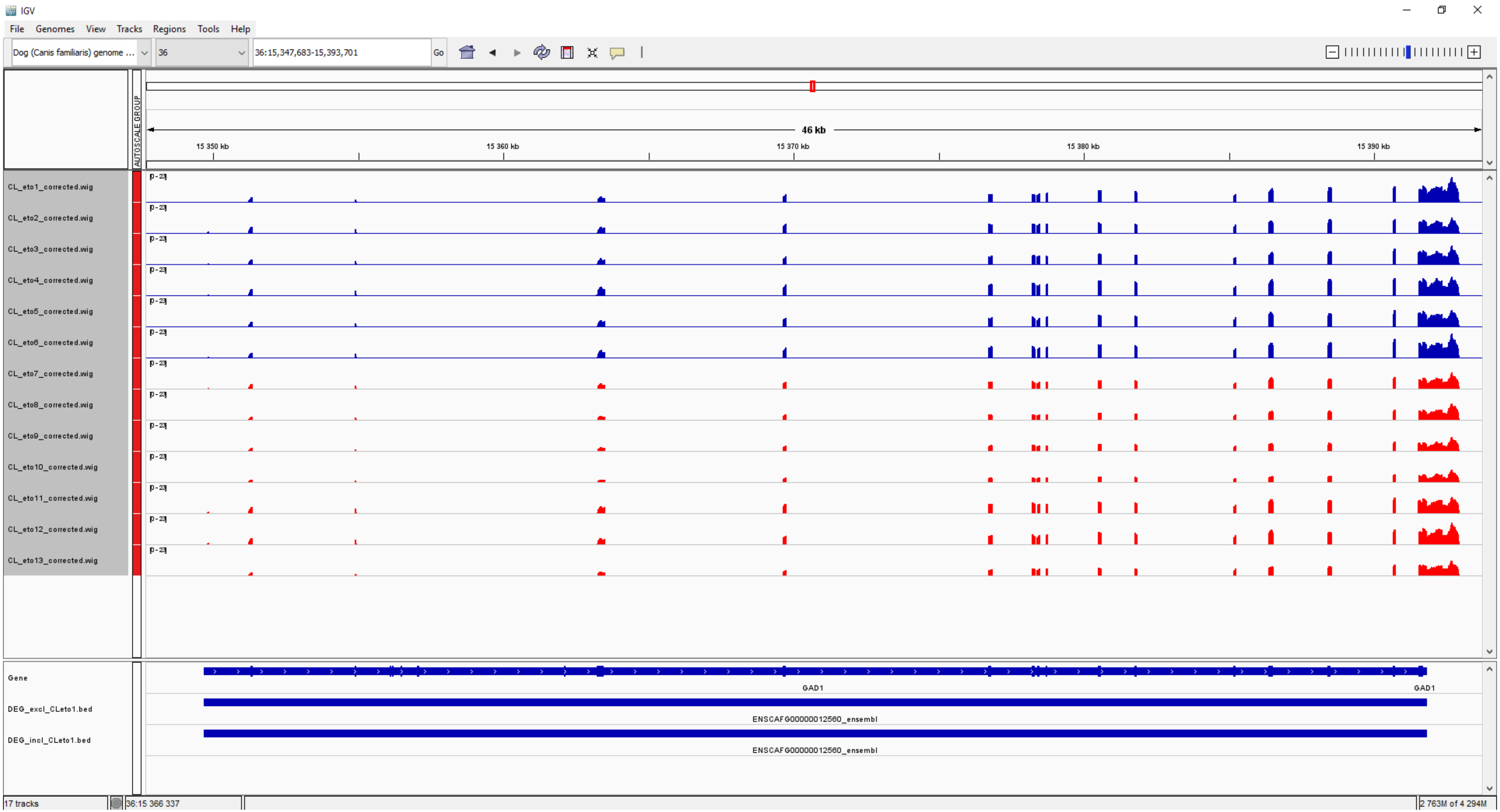

Figure s3

Figure s3: Expression signature for each gene mentioned in detail in the main text. The IGV 2.8.13. software was used for visualization.

g (SST)

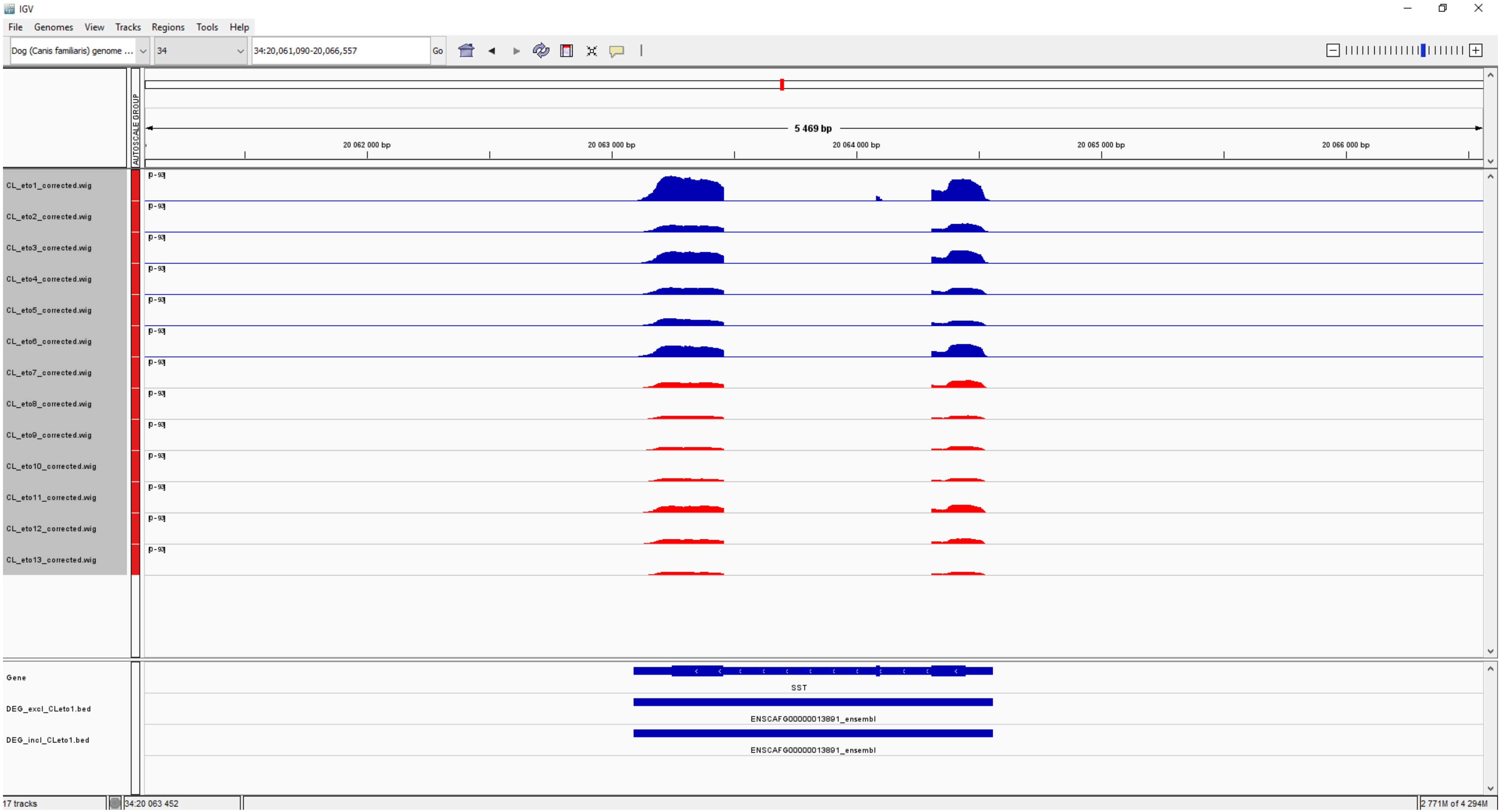

Figure s3

Figure s3: Expression signature for each gene mentioned in detail in the main text. The IGV 2.8.13. software was used for visualization.

h (NPAS4)

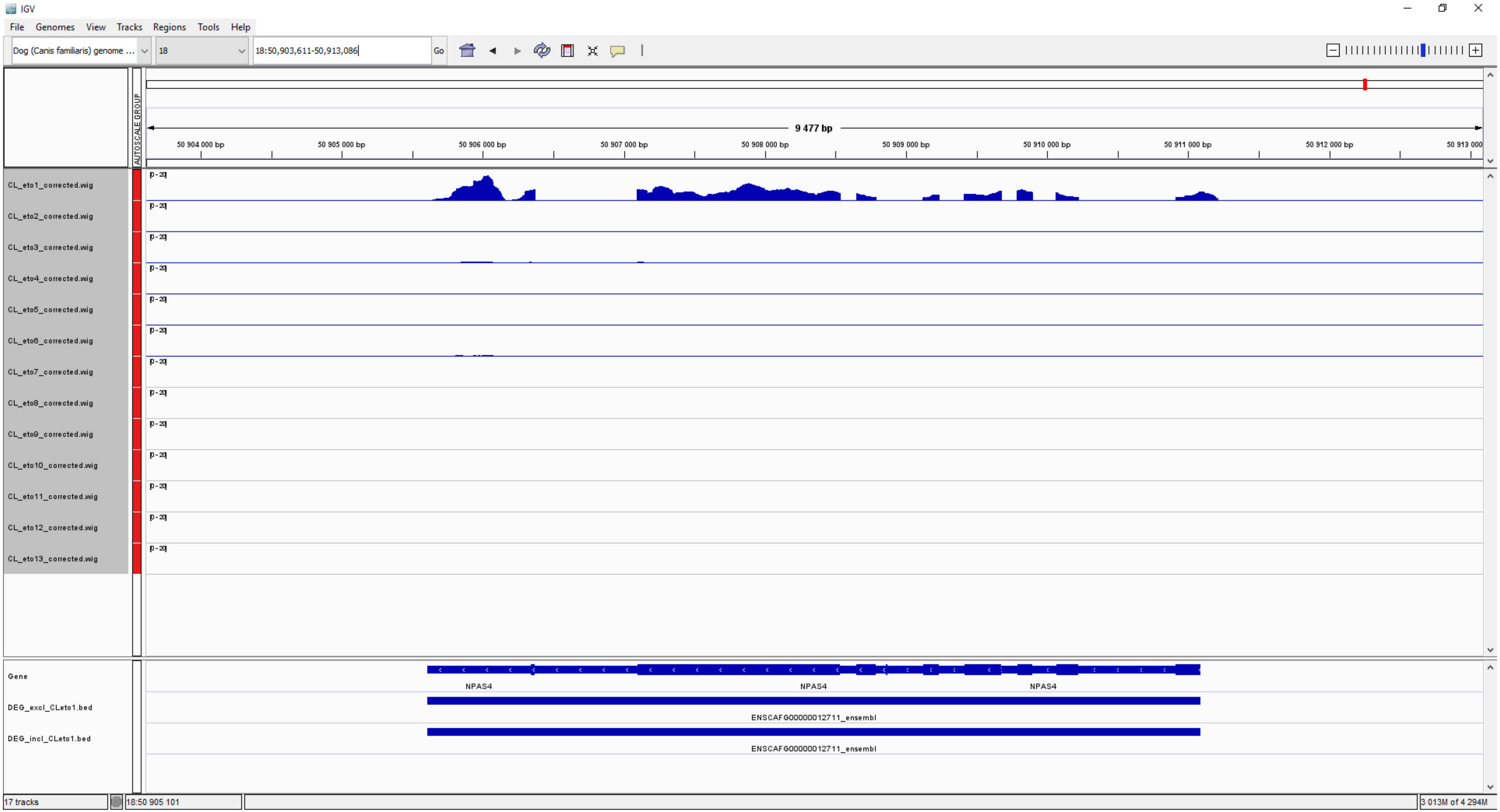

Figure s3

Figure s3: Expression signature for each gene mentioned in detail in the main text. The IGV 2.8.13. software was used for visualization.

i (UPK1B)

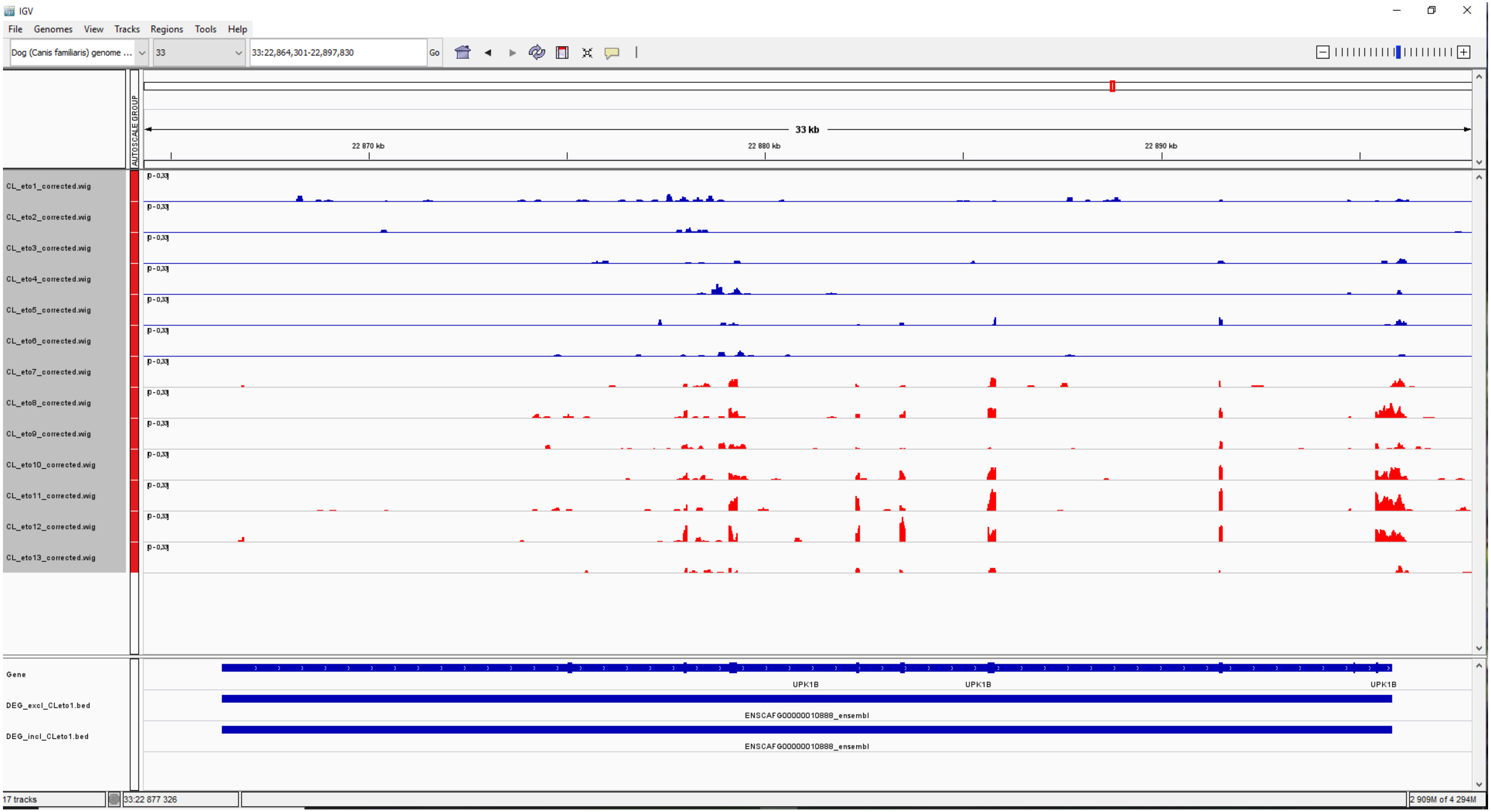

Figure s3

Figure s3: Expression signature for each gene mentioned in detail in the main text. The IGV 2.8.13. software was used for visualization.

j (SLC47A1)

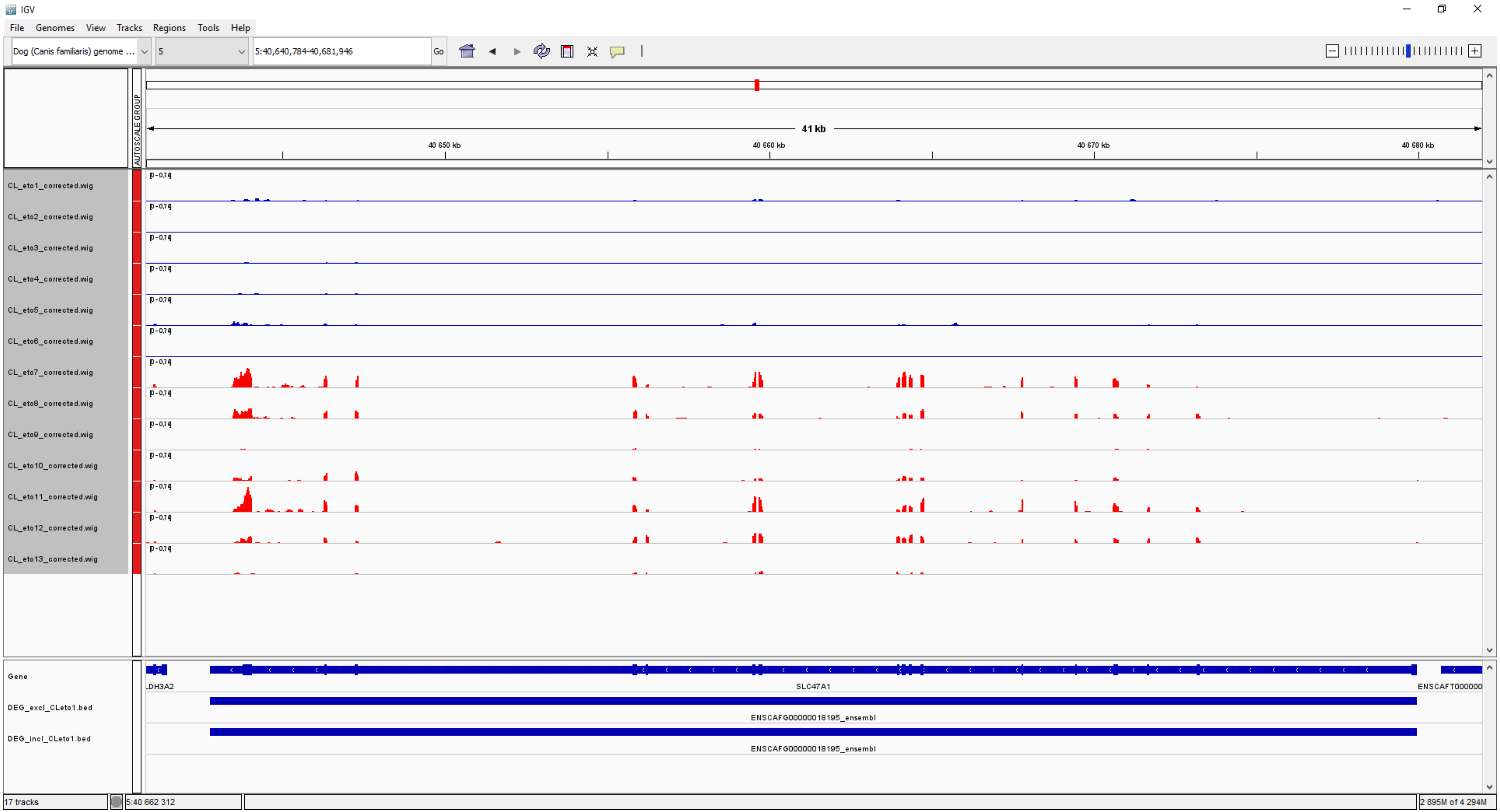

Figure s3

Figure s3: Expression signature for each gene mentioned in detail in the main text. The IGV 2.8.13. software was used for visualization.

k (SERPINE1)

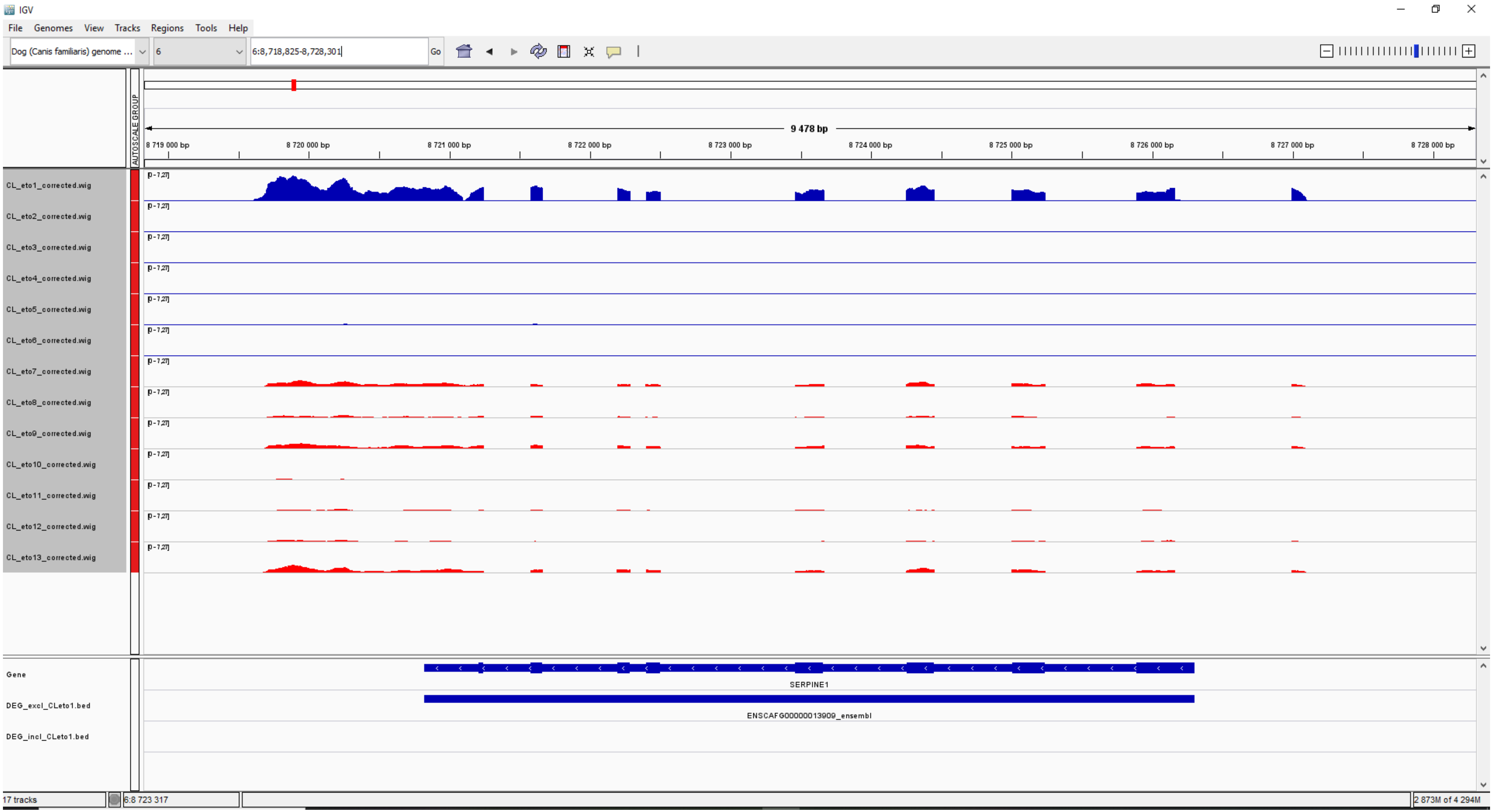

Figure s3

Figure s3: Expression signature for each gene mentioned in detail in the main text. The IGV 2.8.13. software was used for visualization.

I (CCL5)

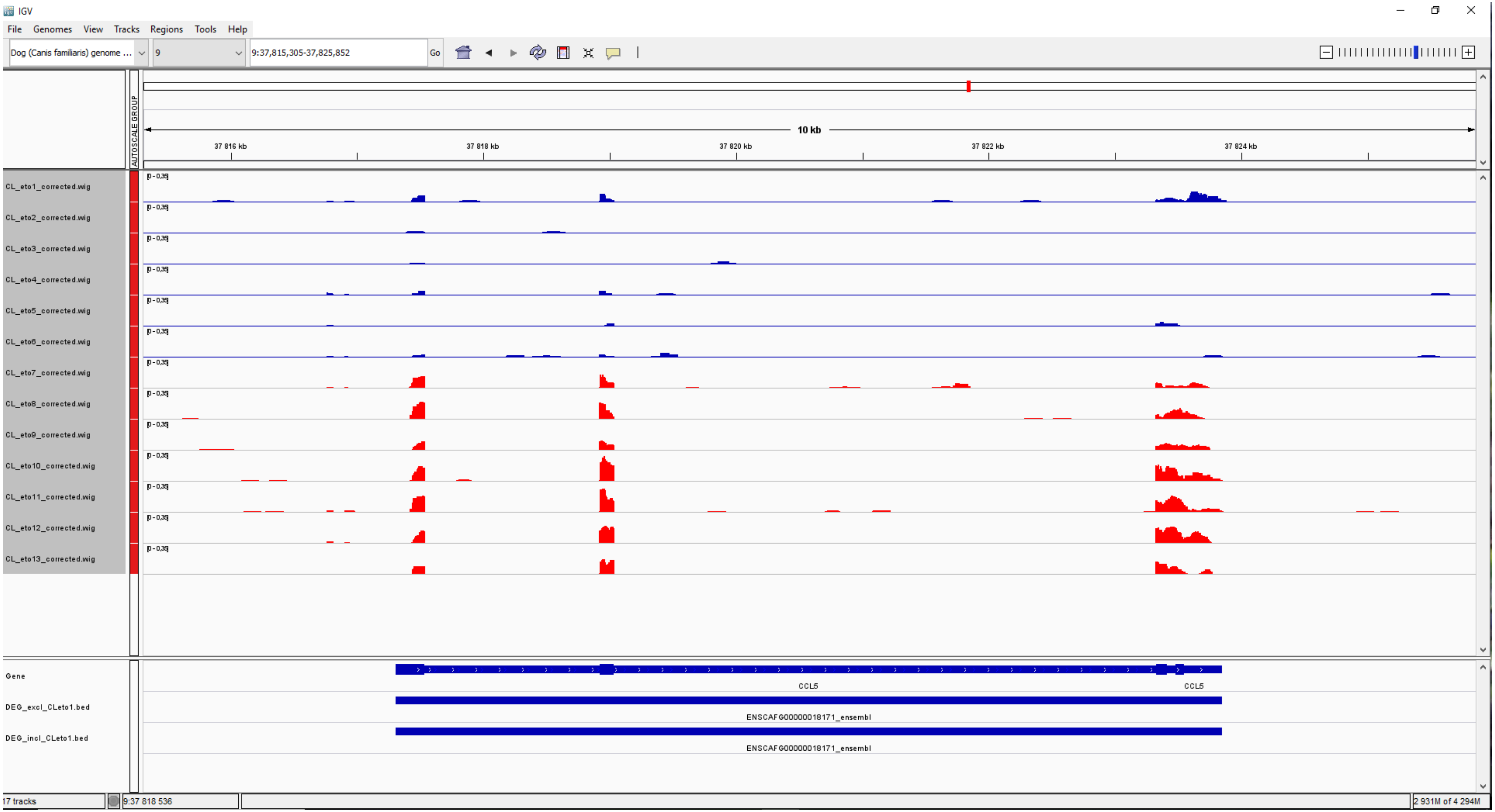

Figure s3

Figure s3: Expression signature for each gene mentioned in detail in the main text. The IGV 2.8.13. software was used for visualization.

m (IBSP)

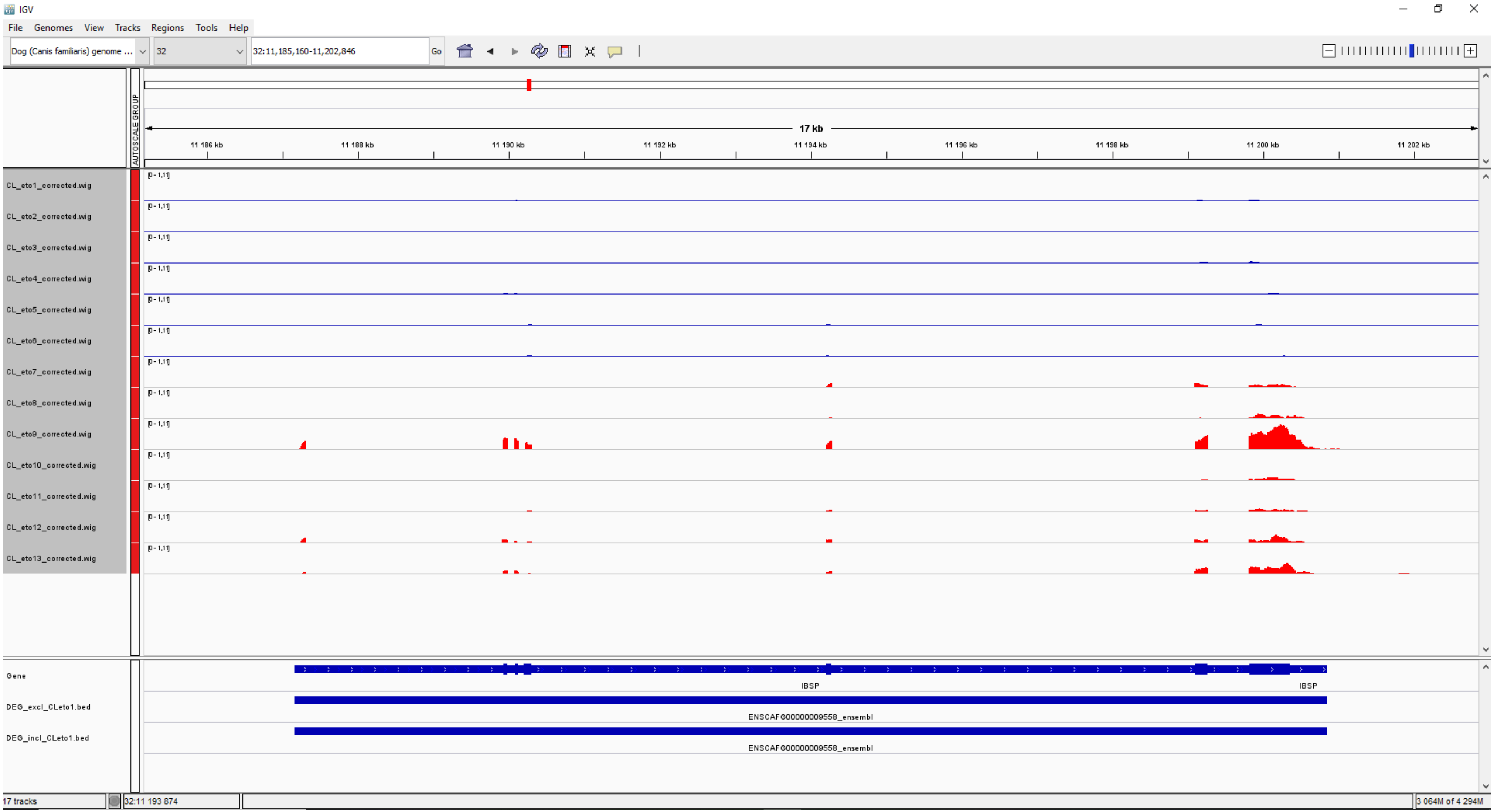

Figure s3

Figure s3: Expression signature for each gene mentioned in detail in the main text. The IGV 2.8.13. software was used for visualization.

n (ENSCAFG00000020220)

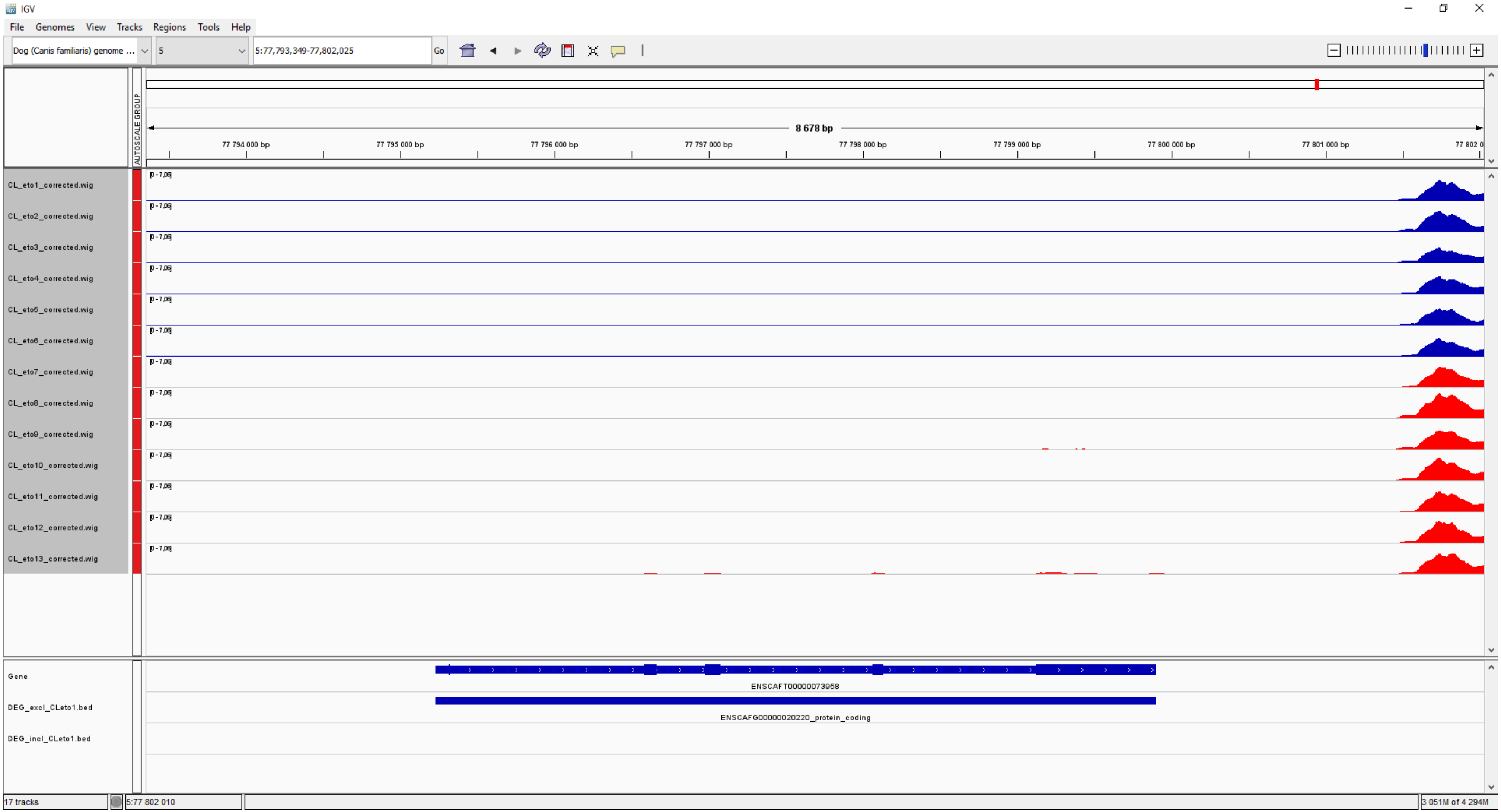

Figure s3

Figure s3: Expression signature for each gene mentioned in detail in the main text. The IGV 2.8.13. software was used for visualization.

o (ECEL1)

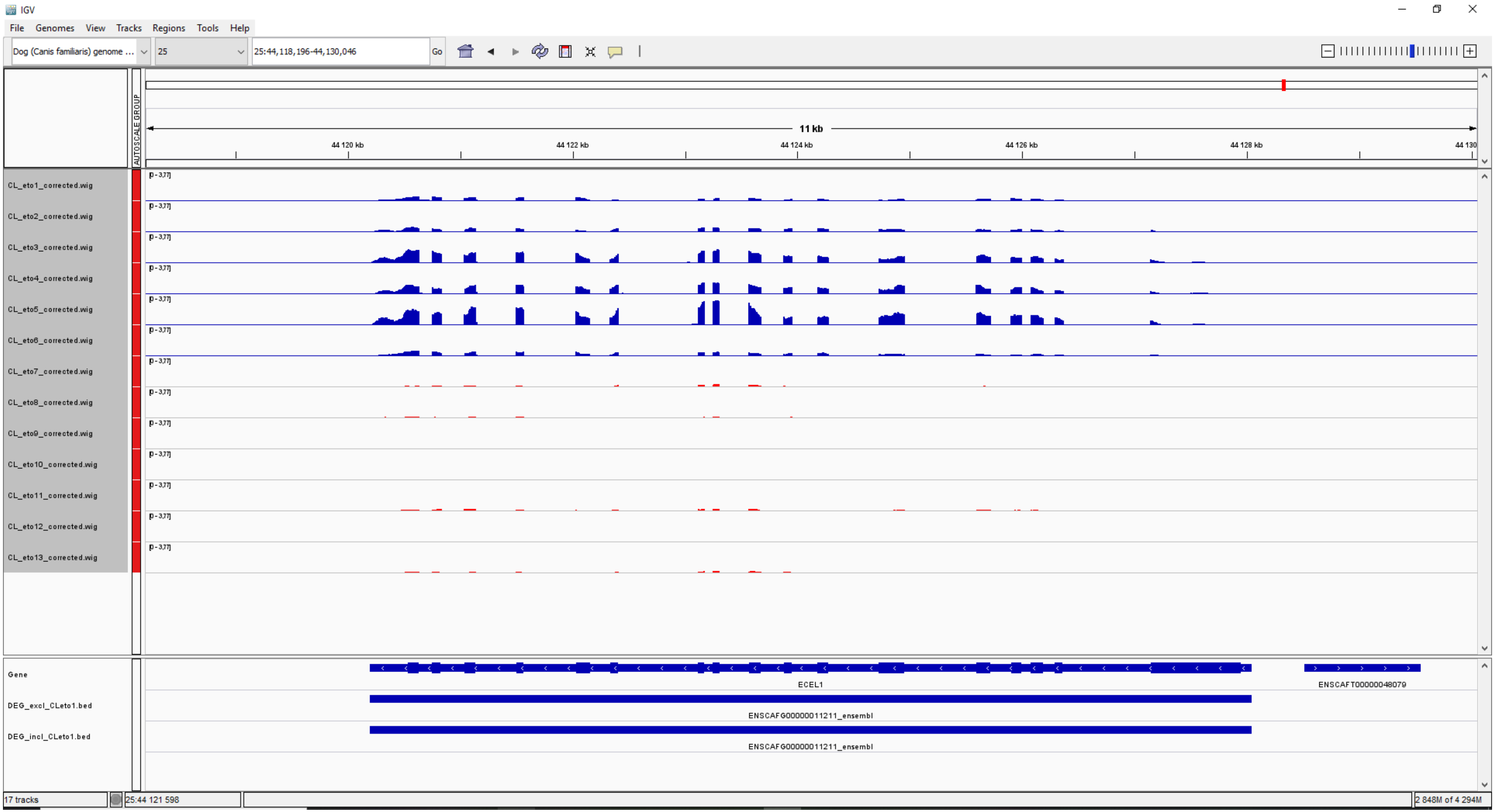

Supplement: Supplementary file 4 — Supplementary file4 (PDF 755 KB) [file 11357_2022_533_MOESM4_ESM.pdf]
