# Supplementary material for: Poly(A) RNA sequencing reveals age-related differences in the prefrontal cortex of dogs
Source: GeroScience. 2022 Mar 14;44(3):1269–93. doi: 10.1007/s11357-022-00533-3 (PMC9213612; doi:10.1007/s11357-022-00533-3)
Supplement: Supplementary file 5 — Supplementary file5 (DOCX 40 KB) [file 11357_2022_533_MOESM5_ESM.docx]

# Supplementary tables

**Table s1.** Primers used in the RT-qPCR experiment.

| **Table S1: Primers used for the real-time quantitative PCR reactions.** | | | |
| --- | --- | --- | --- |
| Oligo name | Sequence | Gene name | Ensembl gene ID |
| Eto_CDKN2A_L | GAGGGCTTCCTGGACACG | CDKN2A | ENSCAFG00000001675 |
| Eto_CDKN2A_R | TCAATTCTTGAAGTCCGGGCT | CDKN2A | ENSCAFG00000001675 |
| Eto_NPAS4_L | CCAGCTGACCACCTAACTG | NPAS4 | ENSCAFG00000012711 |
| Eto_NPAS4_R | AGGTGGATGAGCATGGAATC | NPAS4 | ENSCAFG00000012711 |
| Eto_TNNT2_L | GAAAAGTGGGAAGAGGCAG | TNNT2 | ENSCAFG00000010798 |
| Eto_TNNT2_R | GCTGCTTGAACTTCTCCTG | TNNT2 | ENSCAFG00000010798 |
| Eto_WNT1_L | CTTCGGCAAGATCGTCAAC | WNT1 | ENSCAFG00000008760 |
| Eto_WNT1_R | GATGTTGTCGCTGCAGC | WNT1 | ENSCAFG00000008760 |
| Eto_EGR3_L | CAATCTGTACCCCGAGGAG | EGR3 | ENSCAFG00000009272 |
| Eto_EGR3_R | GGAAGGAGCCCGAATAAGAG | EGR3 | ENSCAFG00000009272 |
| Eto_RSPH1_L | CACAGGTACCAGGGCAAG | RSPH1 | ENSCAFG00000010420 |
| Eto_RSPH1_R | GGAACCAGAGTCATCATCGTC | RSPH1 | ENSCAFG00000010420 |
| Eto_NCCRP1_L | GGGTGGTACATTGGGACTG | NCCRP1 | ENSCAFG00000005593 |
| Eto_NCCRP1_R | CCAGACGTGCAGCTCATAG | NCCRP1 | ENSCAFG00000005593 |
| Eto_DTHD1_L | CTCAGAAGGCCCAAGAGAAG | DTHD1 | ENSCAFG00000016290 |
| Eto_DTHD1_R | CTTGGGGGCTGCTATCAC | DTHD1 | ENSCAFG00000016290 |
| Eto_GFAP_L | CAGCTGCAGACCTTGACC | GFAP | ENSCAFG00000013973 |
| Eto_GFAP_R | GGTTCTGTCCCTCCTCCTC | GFAP | ENSCAFG00000013973 |
| Eto_CD74_L | CATCTCCAACCATGAGCAG | CD74 | ENSCAFG00000018101 |
| Eto_CD74_R | GTGACTGTCAGCTTGTCC | CD74 | ENSCAFG00000018101 |

**Table s2**: The number of differentially expressed genes (DEGs) in 4 different analyses.

| **Analysis** | **Number of DEGs** | **Number of down-regulated genes** | **Number of up-regulated genes** |
| --- | --- | --- | --- |
| **All individuals** | 1152 | 634 | 518 |
| **Excluding CL_eto1** | 3436 | 1701 | 1735 |
| **Excluding CL_eto3** | 878 | 477 | 401 |
| **Excluding CL_eto5** | 668 | 388 | 280 |

**Table s3:** Changes in the number of differentially expressed genes, when either two males or two random animals are left out from the old cohort. „Missing”: the number of DEGs, which are not present in the new list; „Overlap”: the number of genes overlapping between the new and original list; „New”: number of DEGs present in the new list but not in the original one.

| **Permutation** | **DEG number** | **Missing** | **Same** | **New** |
| --- | --- | --- | --- | --- |
| missing_genes_wo2random_v1 | 3326 | 588 | 2848 | 478 |
| missing_genes_wo2random_v2 | 2687 | 942 | 2494 | 193 |
| missing_genes_wo2random_v3 | 2529 | 1058 | 2378 | 151 |
| missing_genes_wo2random_v4 | 3383 | 508 | 2928 | 455 |
| missing_genes_womales | 2475 | 1134 | 2302 | 173 |

**Table s4:** CPM values of four red blood cell specific transcripts.

|  | ENSCAFG00000032615 | ENSCAFG00000029224 | ENSCAFG00000028569 | ENSCAFG00000030286 |
| --- | --- | --- | --- | --- |
| Average | 67.35186 | 0.370188 | NA | 73.65049 |
| CL_eto2 | 3.821414 | 0.464284 | Not expressed | 1.499994 |
| CL_eto3 | 325.613 | 0.65858 | Not expressed | 363.2619 |
| CL_eto4 | 25.93094 | 0.422434 | Not expressed | 26.51585 |
| CL_eto5 | 141.7607 | 0.500921 | Not expressed | 143.6103 |
| CL_eto6 | 51.61822 | 0.501522 | Not expressed | 57.59791 |
| CL_eto7 | 88.06318 | 0.421153 | Not expressed | 102.8878 |
| CL_eto8 | 30.90956 | 0.331648 | Not expressed | 39.10125 |
| CL_eto9 | 5.177713 | 0.376561 | Not expressed | 4.942363 |
| CL_eto10 | 97.47521 | 0.25661 | Not expressed | 103.3406 |
| CL_eto11 | 10.25356 | 0.255427 | Not expressed | 9.998129 |
| CL_eto12 | 5.906625 | 0 | Not expressed | 6.446865 |
| CL_eto13 | 21.69211 | 0.253117 | Not expressed | 24.60295 |

**Table s5.** List of the genes, which overlapped with the Swanson et al (2009) study’s age-related DEG list.

| ABLIM3 | GPHN | PIN1 |
| --- | --- | --- |
| ACTR3 | GPR137 | PKIG |
| AKAP13 | GRB14 | PLK2 |
| ANKRA2 | GSTA4 | PPP2CB |
| ANXA1 | GSTM3 | PRDX6 |
| AP2B1 | HADHA | PTMA |
| APP | HBP1 | PTPRG |
| ATG5 | HDAC3 | QDPR |
| BCL11A | HMGCR | RANBP9 |
| BCL2 | HMGCS1 | RARRES2 |
| BDNF | HMGN3 | RCN1 |
| BPI | IL13RA1 | RDH11 |
| BRD9 | INPP5F | RGS6 |
| C1R | ITPKA | RHOG |
| C3 | KARS | RPL4 |
| CALM3 | KHDRBS3 | RPS21 |
| CAP2 | LAMP1 | RPS25 |
| CAPN1 | LAPTM5 | RRAS2 |
| CASK | LARS | RTN1 |
| CD47 | LMO4 | S100A1 |
| CD74 | LZTS2 | SERBP1 |
| CDH6 | M6PR | SLC17A7 |
| CHD4 | MAP2K4 | SNCA |
| CLSTN1 | MARK1 | SNRPD1 |
| CP | MRC1 | SRPK2 |
| CPE | MTX2 | SST |
| CRH | NELL2 | SSTR2 |
| CRYAB | NFKBIA | SUB1 |
| CTSK | NPC1 | SUMO2 |
| CUEDC2 | NPC2 | TAC1 |
| CXADR | NPY | TLN1 |
| CYBB | NUDC | TLR4 |
| CYCS | NUPR1 | TMEM132A |
| EIF4E | OLFM1 | TXNIP |
| EPHX1 | OSBPL1A | TYRO3 |
| ETFDH | PAK1 | UQCRFS1 |
| FGF13 | PAM | VWF |
| FMNL1 | PCSK1 | WASF1 |
| FTH1 | PDLIM4 | YWHAB |
| GAP43 | PENK | YWHAE |
| GLO1 | PFDN5 | ZNF423 |
| GNB1 | PIK3C3 |  |

**Table s6**: Significantly overrepresented gene ontology terms.

| **GO category** | **GO term** | **Over/under-  represented** | **Fold enrichment** | **FDR** |
| --- | --- | --- | --- | --- |
| **BP** | neuromuscular junction development | + | 14.19 | 0.00028 |
| **BP** | calcium-ion regulated exocytosis | + | 4.14 | 0.01570 |
| **BP** | synaptic vesicle exocytosis | + | 3.55 | 0.01950 |
| **BP** | synaptic transmission. glutamatergic | + | 3.17 | 0.02680 |
| **BP** | stress-activated protein kinase signaling cascade | + | 2.66 | 0.03530 |
| **BP** | modulation of chemical synaptic transmission | + | 2.60 | 0.00356 |
| **BP** | vesicle fusion to plasma membrane | + | 2.18 | 0.01050 |
| **BP** | regulation of membrane potential | + | 2.15 | 0.01450 |
| **BP** | G protein-coupled receptor signaling pathway | + | 1.91 | 0.00084 |
| **BP** | cell-cell adhesion | + | 1.83 | 0.03210 |
| **BP** | regulation of transport | + | 1.71 | 0.02020 |
| **BP** | actin filament organization | + | 1.70 | 0.04490 |
| **BP** | cell morphogenesis | + | 1.64 | 0.03400 |
| **BP** | generation of neurons | + | 1.56 | 0.03000 |
| **BP** | ncRNA processing | - | 0.52 | 0.03370 |
| **BP** | proteasome-mediated ubiquitin-dependent protein catabolic process | - | 0.50 | 0.03340 |
| **BP** | mRNA splicing. via spliceosome | - | 0.50 | 0.03650 |
| **BP** | ATP metabolic process | - | 0.35 | 0.03020 |
| **BP** | DNA biosynthetic process | - | 0.29 | 0.03020 |
| **MF** | glutamate receptor activity | + | 4.19 | 0.01870 |
| **MF** | glutamate binding | + | 4.19 | 0.01760 |
| **MF** | postsynaptic neurotransmitter receptor activity | + | 3.77 | 0.00940 |
| **MF** | calmodulin binding | + | 2.78 | 0.04530 |
| **MF** | neurotransmitter binding | + | 2.42 | 0.01330 |
| **MF** | cell adhesion molecule binding | + | 2.13 | 0.01320 |
| **MF** | molecular adaptor activity | + | 1.99 | 0.03190 |
| **MF** | actin binding | + | 1.77 | 0.03290 |
| **MF** | carbohydrate derivative binding | + | 1.62 | 0.03250 |
| **MF** | sequence-specific DNA binding | - | 0.77 | 0.04390 |
| **MF** | RNA binding | - | 0.62 | 0.00379 |
| **MF** | translation regulator activity | - | 0.33 | 0.04490 |
| **CC** | neuromuscular junction | + | 9.75 | 0.00040 |
| **CC** | leading edge membrane | + | 5.32 | 0.00052 |
| **CC** | presynaptic membrane | + | 5.16 | 0.00041 |
| **CC** | ionotropic glutamate receptor complex | + | 4.73 | 0.00011 |
| **CC** | presynaptic active zone | + | 4.51 | 0.00194 |
| **CC** | axon terminus | + | 3.55 | 0.01400 |
| **CC** | synaptic vesicle membrane | + | 3.34 | 0.00691 |
| **CC** | dendritic spine | + | 3.33 | 0.01010 |
| **CC** | cell division site | + | 3.29 | 0.01980 |
| **CC** | postsynaptic membrane | + | 3.28 | 0.00040 |
| **CC** | postsynaptic density | + | 2.63 | 0.00230 |
| **CC** | cell projection membrane | + | 2.53 | 0.03890 |
| **CC** | actin filament bundle | + | 2.46 | 0.02790 |
| **CC** | cell-substrate junction | + | 2.41 | 0.03910 |
| **CC** | actin filament | + | 2.36 | 0.00165 |
| **CC** | adherens junction | + | 2.36 | 0.00912 |
| **CC** | cation channel complex | + | 2.28 | 0.00110 |
| **CC** | extrinsic component of plasma membrane | + | 2.26 | 0.00969 |
| **CC** | cell cortex | + | 2.03 | 0.00467 |
| **CC** | nuclear chromatin | - | 0.68 | 0.01240 |
| **CC** | nucleoplasm | - | 0.59 | 0.00051 |
| **CC** | spliceosomal snRNP complex | - | 0.42 | 0.04150 |
| **CC** | nucleolus | - | 0.38 | 0.00040 |
| **CC** | nuclear DNA-directed RNA polymerase complex | - | 0.36 | 0.04960 |
| **CC** | integral component of mitochondrial membrane | - | 0.18 | 0.02380 |
| **CC** | mitochondrial respirasome | - | 0.17 | 0.01660 |
| **CC** | inner mitochondrial membrane protein complex | - | 0.13 | 0.00003 |
| **CC** | respiratory chain complex I | - | 0.12 | 0.03890 |
| **CC** | precatalytic spliceosome | - | 0.10 | 0.01360 |
| **CC** | proteasome complex | - | < 0.01 | 0.00055 |

**Table s7**: Gene identifiers of the 26 genes that were found to be differentially expressed in all 3 studies. Abbreviations: MM – Mus musculus; CL – Canis lupus familiaris; HS – Homo sapiens.

| **Gene name** | **MM geneID** | **CL geneID** | **HS gene ID** |
| --- | --- | --- | --- |
| **ARL5A** | ENSMUSG00000036093 | ENSCAFG00000005761 | ENSG00000162980 |
| **C1QB** | ENSMUSG00000036905 | ENSCAFG00000032344 | ENSG00000173369 |
| **C2CD2L** | ENSMUSG00000032120 | ENSCAFG00000012269 | ENSG00000172375 |
| **CEBPA** | ENSMUSG00000034957 | ENSCAFG00000007407 | ENSG00000245848 |
| **CX3CL1** | ENSMUSG00000031778 | ENSCAFG00000008761 | ENSG00000006210 |
| **EPHA4** | ENSMUSG00000026235 | ENSCAFG00000015936 | ENSG00000116106 |
| **GABRA2** | ENSMUSG00000000560 | ENSCAFG00000025069 | ENSG00000151834 |
| **HDAC2** | ENSMUSG00000019777 | ENSCAFG00000004049 | ENSG00000196591 |
| **HIVEP2** | ENSMUSG00000015501 | ENSCAFG00000000300 | ENSG00000010818 |
| **HOMER1** | ENSMUSG00000007617 | ENSCAFG00000009074 | ENSG00000152413 |
| **ITM2C** | ENSMUSG00000026223 | ENSCAFG00000028707 | ENSG00000135916 |
| **KIAA1644** | ENSMUSG00000062760 | ENSCAFG00000000873 | ENSG00000138944 |
| **LANCL1** | ENSMUSG00000026000 | ENSCAFG00000014027 | ENSG00000115365 |
| **NRP1** | ENSMUSG00000025810 | ENSCAFG00000003776 | ENSG00000099250 |
| **PML** | ENSMUSG00000036986 | ENSCAFG00000025384 | ENSG00000140464 |
| **PRKCD** | ENSMUSG00000021948 | ENSCAFG00000008689 | ENSG00000163932 |
| **PTGR1** | ENSMUSG00000028378 | ENSCAFG00000003000 | ENSG00000106853 |
| **SCG2** | ENSMUSG00000050711 | ENSCAFG00000016186 | ENSG00000171951 |
| **SCG3** | ENSMUSG00000032181 | ENSCAFG00000015489 | ENSG00000104112 |
| **SEMA4A** | ENSMUSG00000028064 | ENSCAFG00000016857 | ENSG00000196189 |
| **SLC8A2** | ENSMUSG00000030376 | ENSCAFG00000004140 | ENSG00000118160 |
| **SORCS1** | ENSMUSG00000043531 | ENSCAFG00000010628 | ENSG00000108018 |
| **STMN1** | ENSMUSG00000028832 | ENSCAFG00000029191 | ENSG00000117632 |
| **STMN3** | ENSMUSG00000027581 | ENSCAFG00000013020 | ENSG00000197457 |
| **THBS3** | ENSMUSG00000028047 | ENSCAFG00000017017 | ENSG00000169231 |
| **TRIM24** | ENSMUSG00000029833 | ENSCAFG00000024739 | ENSG00000122779 |

**Table s8**: RT-qPCR and RNA-Seq detected fold changes of 10 genes chosen for validation of the results.

| **Gene** | **RNA-Seq direction** | **PCR direction** | **PCR fold change** | **RNA-Seq fold change** |
| --- | --- | --- | --- | --- |
| **CDKN2A** | UP | UP | 5,36 | 3,58 |
| **NCCRP1** | UP | UP | 7,99 | 8,25 |
| **DTHD1** | UP | UP | 4,03 | 3,82 |
| **GFAP** | UP | UP | 5,31 | 3,71 |
| **CD74** | UP | UP | 6,65 | 4,30 |
| **NPAS4** | DOWN | DOWN | 0,45 | 0,23 |
| **TNNT2** | DOWN | DOWN | 0,06 | 0,05 |
| **WNT1** | DOWN | DOWN | 0,83 | 0,63 |
| **EGR3** | DOWN | DOWN | 0,65 | 0,60 |
| **RSPH1** | DOWN | DOWN | 0,26 | 0,27 |
